# Exploring magnetoelectric nanoparticles for advanced nano-electroporation and drug delivery in interventional cardiology

**DOI:** 10.1039/d5na00438a

**Published:** 2025-08-22

**Authors:** A. Tommasini, G. Suarato, S. Fiocchi, E. Chiaramello, A. Marrella, M. Lenzuni, M. Parazzini, B. Cortese, P. Ravazzani

**Affiliations:** a Dipartimento di Elettronica, Informazione e Bioingegneria, Politecnico di Milano Piazza Leonardo da Vinci 32 Milano 20133 Italy; b Cnr-Istituto di Elettronica e di Ingegneria Dell’Informazione e Delle Telecomunicazioni, Piazza Leonardo da Vinci 32 Milano 20133 Italy giulia.suarato@cnr.it; c Interventional Coronary Center, University Hospitals Harrington Heart & Vascular Institute Cleveland Ohio USA; d Fondazione Ricerca e Innovazione Cardiovascolare Via E. Ponti, 49 Milan 20136 Italy; e DCB Academy Via E. Ponti, 49 Milan 20136 Italy

## Abstract

Leveraging their ability to induce an intense and highly localized electric polarization upon application of an external magnetic field, core–shell magnetoelectric nanoparticles (MENPs) constitute powerful nanotools to achieve wireless nano-electroporation and targeted drug delivery in critical applications such as cardiovascular medicine, where frequent interventions may pose general yet high risks to the patients. In this study, the magnetoelectric feature is exploited to thoroughly map *in silico* the behavior of a model MENP under a set of either static or time-varying external magnetic fields, with the dual aim of (i) providing a series of operational parameters to elicit at the nanoscale a reversible cell membrane poration and (ii) correlating the electric potential difference developed at the activated MENP surface with the charge displacement potentially induced in a hypothetical drug-MENP bond. The finite element analysis framework considers a single cobalt ferrite core–barium titanate shell MENP, either immersed in culture medium or engulfed in a blood vessel wall. Simulation results obtained under static magnetic field conditions show that maximizing magnetoelectric efficiency (79.82 mV Oe^−1^ cm^−1^) generates electric fields in line with reversible nano-electroporation values (3.7 × 10^4^ to 4.7 × 10^4^ V m^−1^). Furthermore, a correlation between the profiles of the electric potential registered at the MENP outer border (2.86–2.92 mV) and the charge displacement hypothetically experienced by a drug-MENP bond is highlighted, when an alternating, low-intensity external magnetic field is used. Our computational study sets a preliminary base to gain a deeper understanding of the interface phenomena of MENP-mediated cell poration and drug delivery, and it serves as a preliminary step towards complex future analyses.

## Abstract 2

Magnetoelectric nanoparticles can develop electric fields at their surface and in their surroundings, upon remote activation *via* a human-safe magnetic field. Such feature is explored to design innovative strategies for on-demand drug delivery.

## Graphical abstract

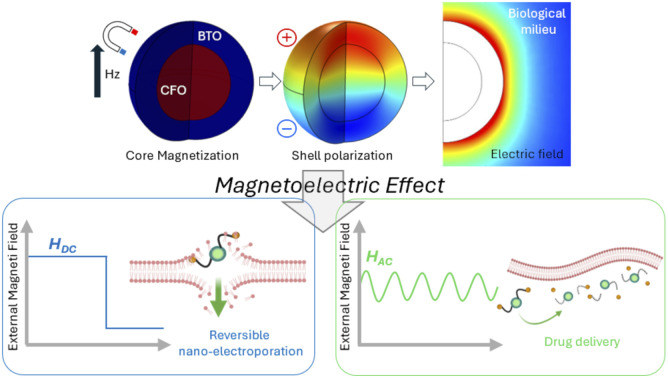

## 1. Introduction

Despite advances in medicine, particularly in the cardiovascular field, restenosis remains a common and significant problem following coronary artery revascularization. Pathologies, such as atherosclerotic plaques and cardiac ischemia, often require the use of stents to restore vessel patency and physiological blood circulation.^1^ However, the incidence of in-stent restenosis (ISR), *i.e.* a progressive reduction in the vessel lumen size (reaching more than 50%) may be induced by an upregulated repair response, increases patient morbidity and may also cause acute myocardial infarction.^2^ Restenosis occurs when a vessel, usually a coronary artery, is damaged after percutaneous revascularization. The process that follows this event involves several interrelated mechanisms, and the trauma results in vascular injury, triggering a local inflammatory response and the production of cytokines and growth factors at the damaged site.^3^ The presence of these molecules causes vascular smooth muscle cells (VSMCs) to migrate into the innermost layer of the vessel, where they proliferate excessively. VSMCs begin to produce large amounts of extracellular matrix (ECM), consisting of collagen and proteoglycans, which gradually lead to the occlusion of the vessel lumen. Such ECM deposition concurs with vessel remodeling and, in parallel, thrombus formation on the damaged vessel surface, which further contributes to the restenosis process.^4^

To tackle this medical issue, in the late 1970s and mid-1980s Dotter and Judkins performed the first coronary angioplasty in a patient with a bare metal stent.^5^ Later, bare metal devices were replaced by drug-eluting stents, approved by the Food and Drug Administration in 2003,^6^ which improved physiological revascularization and lowered the mortality rate. Despite optimization in stent design, the use of thinner struts and lightweight materials, or the research on more biocompatible coatings^7^ to further sustain the therapeutics release, persistent inflammation around the implanted stent and delayed endothelial repair remain critical in ISR.^8^ Later, a new class of devices has been developed, namely drug-coated balloons, which allow drug delivery without prosthesis implantation.^9,10^ This novel medical apparatus was designed to initially tackle ISR and later showed promising results also for the treatment of native coronary artery disease.^11^

To this end, nanotechnology can offer a variety of curative approaches, especially when it comes to engineering platforms for targeted drug delivery. For example, micro- and nanoparticles can find applications in a plethora of different medical fields (*i.e.*, tissue engineering, wound healing, and anticancer therapy, to name a few) due to their variety in terms of sizes, organic and inorganic components,^12,13^ morphologies, and fabrication and functionalization techniques.^14,15^ By tuning the surface chemistry of these nanosystems, site-specific and cellular-targeted delivery can be achieved^16^ and the drug loading content optimized,^17^ while challenges such as poor pharmacokinetics, drug instability under physiological conditions, or enzymatic degradation can be limited by properly combining cargo molecules with nanoparticle carriers.^18^ Leveraging their therapeutic potential, micro- and nano-polymeric particles or lipid-based micelles have been recently exploited for cardiovascular applications.^7,19–21^ To achieve a more precise spatial directionality towards a stenotic vessel region, magnetic nanoparticles, properly decorated with polymeric coatings and active drugs, have been locally and systemically administered to mice and subjected to an external static magnetic field. For example, Fellows *et al.*^22^ delivered heparin-coated magnetic nanoparticles for drug therapy of neointimal hyperplasia, while Polyak and co-workers^23^ injected endothelial cells previously loaded with superparamagnetic nanoparticles, to allow for their blood navigation and targeted positioning near a stented area to inhibit ISR.

In addition to these more conventional systems, a novel class of multi-functional nanostructures is represented by magnetoelectric materials in which magnetic and electric fields can be strongly coupled at body temperature.^24^ More specifically, magnetoelectric nanoparticles (MENPs) have recently emerged as a promising frontier in biomedical research due to their unique ability to act as nanoconverters.^25^ In fact, these multi-phase structures are based on the combination of a magnetostrictive core and a piezoelectric shell, with different sizes and shapes, whose interface coupling can be optimized^26^ to achieve desired features and performance.^27^ Thanks to their ability to polarize and generate an intense electric field at their surface (order of magnitude of 10^5^ V m^−1^) and in their immediate nearby, MENPs have been investigated for (i) wireless brain and nervous tissue stimulation,^28–31^ (ii) cell manipulation,^32^ (iii) cancer treatment, through hyperthermia or targeting of cancer cells,^33,34^ (iv) tissue engineering,^35^ and (v) nano-electroporation and drug delivery.^36–38^

Classical electroporation methods use traditional electrodes to generate high-intensity electric fields for the permeabilization of cell membranes. However, such electrodes are often invasive and difficult to apply in *in vivo* settings.^39,40^ Despite its efficacy, the application of a high intensity, non-localized electric field can lead to difficult and non-specific control of the process and even to loss of function or cell death (irreversible nano-electroporation). Greater spatial control of the generated electric field and high versatility in nano-electrode design can be achieved by using MENPs, which could act, when activated with a direct current magnetic field (*H*_DC_), as electric field nano-generators within safe ranges (2 × 10^4^ to 5 × 10^4^ V m^−1^),^41^ able to provide reversible and wireless cell membrane dielectric breakdown.^42^ Similarly, conventional approaches to delivering drugs must contend with poor specificity and loss of drug concentration, as well as difficulties in crossing biological barriers. MENPs can be easily and highly functionalized, as reported in extensive *in vitro* studies by Guduru *et al.*,^43^ where nanoparticles were loaded with paclitaxel as a mitotic inhibitor for ovarian cancer treatment and labelled with a biomarker-specific antibody, and by Nair and coworkers,^44^ who loaded MENPs with an anti-HIV compound. MENPs can achieve drug release following remote activation *via* alternating current, low-intensity magnetic fields (*H*_AC_),^44^ thus providing a field-controlled and highly specific delivery to the diseased tissue. Experimentally, the MENP-mediated drug release has been achieved with the application of an external *H*_AC_ of small magnitude (approximately below 50 Oe, with a frequency ranging from 10 to 100 Hz^36,37^), which was sufficient to liberate a significant drug concentration into the cells.

By combining their ability to electroporate biological membranes with nanometric precision (when subjected to a *H*_DC_ field) alongside the possibility of remotely modulating their cargo molecule unloading (in the presence of a *H*_AC_ field), MENPs appear as a fascinating alternative to target pathological issues also in a yet unexplored field, such as interventional cardiology. In fact, the risk of restenosis and the need for further interventions could be tremendously reduced by employing MENPs, properly decorated with anti-inflammatory or anti-proliferative drugs.

**Fig. 1:**
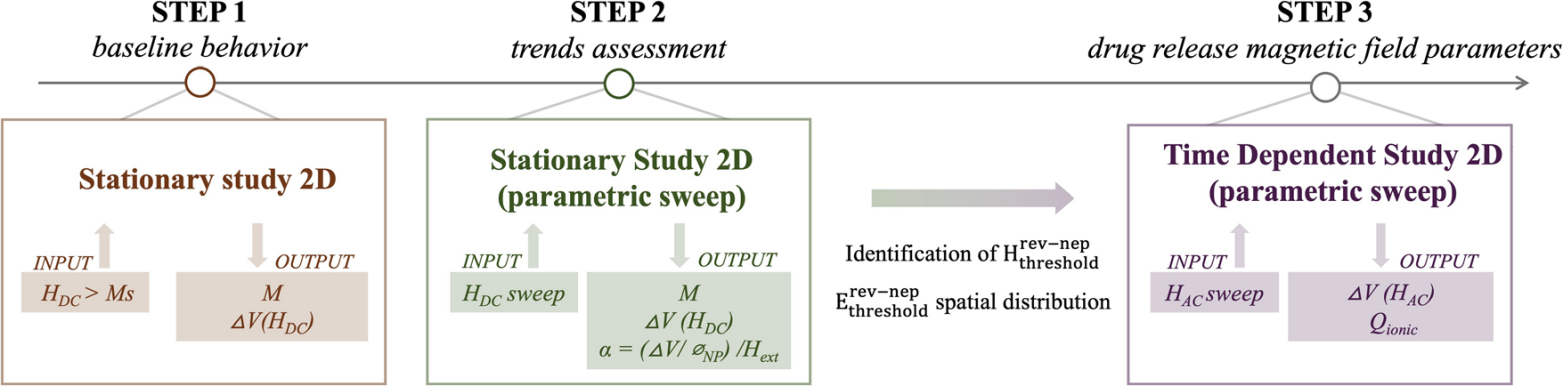
MENP computational study workflow. Step 1: for the baseline behavior a static magnetic field (*H*_DC_) above the material magnetization saturation (*M*_s_) is used as input, while core magnetization (*M*) and the electric potential difference at the MENP surface (Δ*V*(*H*_DC_)) are the output quantities. Step 2: for the sweeping stationary study, varying static magnetic fields (*H*_DC_) are applied and core magnetization (M), electric potential difference Δ*V*(*H*_DC_), and magnetoelectric (ME) coefficient *α* are derived. This step is essential to identify *H*_threshold_^Rev-Nep^, which defines the minimum magnetic field required to generate an electric field (*E*_threshold_^Rev-Nep^) for the reversible nano-electroporation phenomenon to occur. Step 3: in the time dependent study, varying external, low-intensity alternating magnetic fields (*H*_AC_) are imposed to derive the operational parameters governing the drug release process involved in a hypothetical drug-MENP ionic bond breaking. Outputs of step 3 are the electric potential Δ*V*(*H*_AC_) generated at the MENP outer shell and the term *Q*_ionic_, which represents the charge displacement of a hypothetical drug-MENP ionic bond.

With the aim of assessing the feasibility and functionality of such advanced nano-systems for cardiovascular drug release applications, the present study uses an *in silico* computational approach. A preliminary assessment of the MENP baseline behavior when subjected to an external magnetic field above its core magnetization saturation (*M*_s_) was carried out to validate the soundness of our computational model and confirm the presence of the magnetoelectric effect (Fig. 1, step 1). Next, a series of simulations were carried out considering a single nanoparticle immersed in two different biological surroundings, such as the culture medium and the blood vessel wall tissue, exposed to magnetic fields of varying waveforms and intensities. A first parametric-sweep study under a static magnetic field (*H*_DC_) (Fig. 1, step 2) was envisioned to define the operational ranges of MENP stimulation able to subsequently induce reversible membrane permeability. Afterwards, the external magnetic field assumes an alternating pattern (*H*_AC_) (Fig. 1, step 3), which causes an oscillation of the generated potential difference across the nanoparticle, a phenomenon that has been hypothesized to be related to drug bond weakening and, consequently, drug release. The workflow reported in Fig. 1 summarizes the computational steps, highlighting the operational parameters considered in the analysis.

## 2. Materials and methods

### 2.1 Nanoparticle modeling

**Fig. 2:**
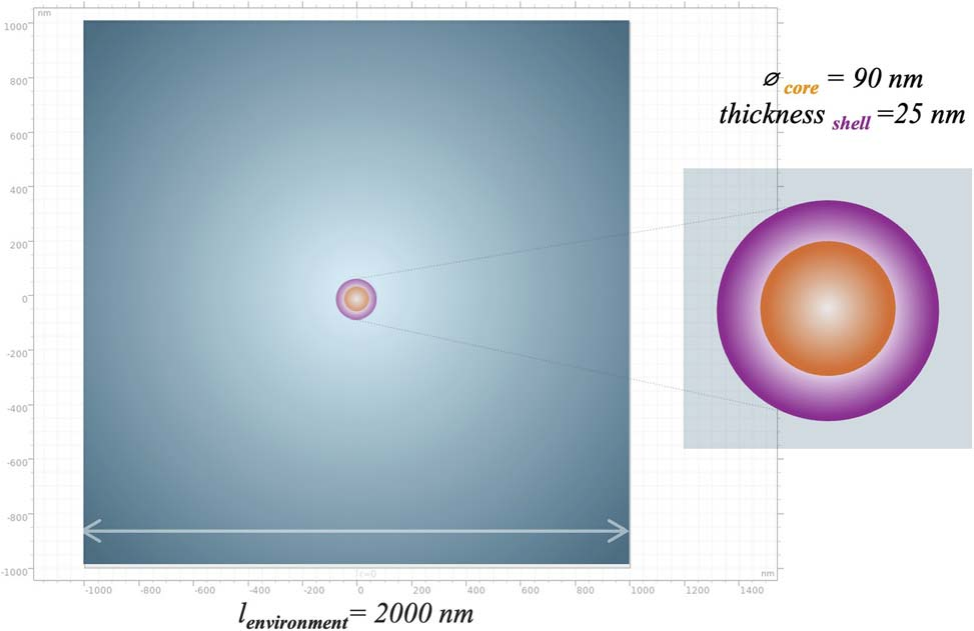
Computational model of the core–shell magnetoelectric nanoparticle, immersed in the surrounding material, with sizes of the geometry.

COMSOL Multiphysics® 5.6 (https://www.comsol.com), a computational tool based on the finite element approach, was adopted as the simulation environment to study the magnetoelectric response of a single, core–shell structured MENP, by means of an axisymmetric bi-dimensional (2D) model. As shown in Fig. 2, the system under study was composed of three domains: (i) a piezoelectric barium titanate shell (BaTiO_3_, in the following referred to as “BTO”), presenting a uniform thickness of 25 nm; (ii) a magnetostrictive cobalt ferrite core (CoFe_2_O_4_, in the following referred to as “CFO”), with a diameter of 90 nm; and (iii) a surrounding environment defined as a squared domain with a side length of 2000 nm. The properties of the shell and core materials, mostly derived from experimental studies and the COMSOL Multiphysics® 5.6 integrated library, are reported in Tables 1 and 2, respectively.

**Table 1:** Barium titanate shell properties

Property	Symbol	Value	Reference
Density (kg m^−3^)	*ρ*	5.2 × 10^3^	45, COMSOL library
Relative permittivity	*ε*_r_	10	COMSOL library
Electrical conductivity (S m^−1^)	*σ*	5.2 × 10^6^	COMSOL library
Poisson's ratio	*ν*	0.48	46, COMSOL library
Young's modulus (Pa)	*E*	230 × 10^9^	46 and 47
Saturation magnetization (A m^−1^)	*M*_s_	3.69 × 10^5^	48
Initial magnetic susceptibility	*χ*_0_	3	49, COMSOL library
Saturation magnetostriction (ppm)	λ	−200	42 and 46

**Table 2:** Cobalt ferrite core properties

Property	Symbol	Culture medium		Blood vessel wall	
		Value	Reference	Value	Reference
Electric conductivity (S m^−1^) 76 543	*σ*	1.5	50	0.232	51
Relative permittivity	*ε*_r_	1	—	1	—
Relative permeability	*μ*_r_	1	—	1	—
Young's modulus (MPa)	*E*	—	—	1	52
Poisson's ratio	*ν*	—	—	0.49	52
Density (kg m^−3^)	*ρ*	1007	51	1102	51

Two different scenarios were modeled, based on the environment in which the nanoparticle was embedded: (i) single MENP floating in the culture medium in a Petri dish; (ii) single MENP enclosed in the blood vessel wall. In both simulations, the surrounding environments were considered as purely resistive materials, with a relative permittivity (*ε*_r_) of 1; the culture medium was modelled as a liquid environment, while the blood vessel was considered a solid material (properties reported in Table 3).

**Table 3:** Properties of the surrounding materials considered in the different simulation scenarios

Property	Symbol	Culture medium		Blood vessel wall	
		Value	Reference	Value	Reference
Electric conductivity (S m^−1^)	*σ*	1.5	50	0.232	51
Relative permittivity	*ε*_r_	1	—	1	—
Relative permeability	*μ*_r_	1	—	1	—
Young's modulus (MPa)	*E*	—	—	1	52
Poisson's ratio	*ν*	—	—	0.49	52
Density (kg m^−3^)	*ρ*	1007	51	1102	51

### 2.2 Stationary study

Our investigation was conducted in three main phases, as outlined in the workflow of Fig. 1. In the first phase, a stationary study (*i.e.*, the MENP was subjected to a high-intensity external static magnetic field, such as 2T, above the core magnetization saturation) was performed to evaluate the baseline magnetoelectric behavior of the MENP within the two distinct environments. Both environments were treated as purely resistive materials with the relative permittivity (*ε*_r_) set to 1. For the culture medium, the electrical conductivity (*σ*) was set to 1.5 S m^−1^, as found in the literature;^53^ while for the blood vessel wall, the electrical conductivity was set to 0.232 S m^−1^, as reported in the IT’IS Foundation's Virtual Population Tissue Properties Database.^51^ Additionally, for the case of the blood vessel wall, the surrounding was modeled as a solid material, characterized by mechanical properties such as Young's modulus, Poisson's ratio and density, as detailed in Table 3. Following the validated computational design previously published by our group,^27,31,34^*in silico* MENP activation was obtained by implementing three different COMSOL Multiphysics® 5.6 modules, such as Magnetic Fields, Solid Mechanics, and Electrostatics, while the Multiphysics mode allowed for the coupling of Magnetostriction and Piezoelectric physics for a complete simulation of the physical phenomenon. The mathematical equations governing the magnetoelectric coupled model have been thoroughly described by Fiocchi *et al.*^54^ and briefly summarized in the SI.

### 2.3 Sweep study – static external magnetic field

In the second step of the study, the magnetoelectric behavior of a single MENP subjected to a wider range of static magnetic field values was assessed. In the *Study* section of the COMSOL Multiphysics® 5.6 interface, the *Stationary Study* was added, and a parametric sweep was selected. The imposed external magnetic field was varied from 0 to 4 T, considering the following intervals: 2–50 mT, 100–800 mT, and 1–4 T. The “*entry method*” of range was set on the “*number of values*”, and fifteen values of each sub-interval were analyzed. Particular attention was given to key parameters, such as (i) the degree of magnetization of the CFO core, (ii) the electric potential generated on the BTO shell surface, and (iii) the associated ME coefficient. The latter is defined according to the equation 
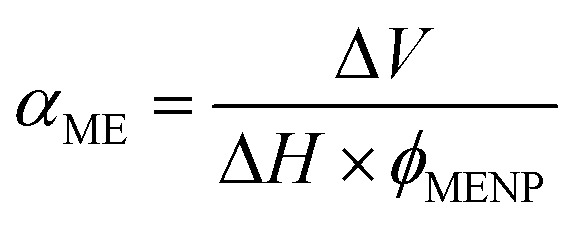
, as the ratio between the electric potential difference on the MENP surface (Δ*V*), expressed in mV, and the product of the applied external magnetic field (*H*), expressed in Oe, and the MENP diameter (*ϕ*_MENP_), given in cm.

At this step of the investigation, particular attention was paid to the electric field generated by the particle within its surroundings. To do so, three representative values of the external magnetic field (*H*_ext_) were considered for the triggering of the magnetoelectric phenomenon and the following data analysis: (1) *H*_ext(1)_ identified in the range of linear dependence between *α*_ME_ and the magnetic field; (2) *H*_ext(2)_ corresponding to the maximum *α*_ME_ with the aim of taking advantage of the optimal MENP performance; (3) *H*_ext(3)_ corresponding to the minimum magnetic field yielding the highest electric potential. In particular, *H*_ext(3)_ was identified as the value of the magnetic field at which the increment between two consecutive potential differences was less than 0.15 mV. A MATLAB code was implemented to examine the near surroundings of the nanoparticle starting from the electric field data distribution exported from COMSOL software. The regions of interest were modeled as concentric, circular semi-coronas around the nanoparticle, identifying areas within three different, increasing distances from the MENP surface. More specifically, the radii of the semi-coronas considered for the analysis were 75, 80 and 90 nm to sample electric field values located at distances of 5, 10 and 20 nm, respectively, from the shell external border (*r*_MENP_ = 70 nm). For each configuration, the electric field distribution was visualized, and its statistical metrics (in terms of median value and the 1^st^ and the 99^th^ percentiles) were extracted to allow for data comparison. More specifically, three regions were considered, depending on how likely the generated electric fields could cause Irreversible Nano-electroporation (Ir-Nep), Reversible Nano-electroporation (Rev-Nep) or none of the two. Such a tendency was assessed by checking the 1^st^ and 99^th^ percentiles of each distribution. The analysis was conducted for the two case scenarios presented in paragraph 2.1 (*i.e.*, culture medium and blood vessel wall). The outputs were processed *via* OriginPro 2023 (OriginLab, United States) and presented as box plots, highlighting median values and percentiles. As summarized in Fig. 1, the aim of this intermediate step was to identify the optimal magnetic field (*H*_threshold_^Rev-Nep^) upon activation of which an electric field comparable to those used in conventional reversible electroporation (*E*_threshold_^Rev-Nep^) could be developed in the immediate vicinity of the MENP. Such *H*_field_ and *E*_field_ and their spatial distributions will be essential for the last step of the investigation.

### 2.4 Sweep study – time-dependent analysis

**Fig. 3:**
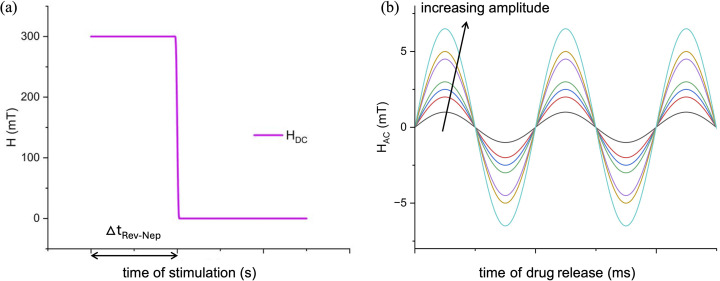
Computational stimulation settings of the time-dependent sweep study: external *H*_field_ profiles. (a) In the first phase (Δ*t*_Rev-Nep_ = time of nano-electroporation), a static magnetic field with a constant amplitude phase (*H*_DC_ = 300 mT) is applied, to allow for a reversible cellular nano-electroporation, while in the following step (b) (Δ*t*_dr_ = time of drug release) an external magnetic field presenting a sinusoidal phase with variable amplitudes (*H*_AC_) is activated (keeping constant the frequency at 100 Hz), to favor the hypothetical drug-MENP bond loosening, initiating the drug delivery process.

Lastly, a time dependent study was performed, with the aim of analyzing the magnetoelectric behavior of the nanoparticle exposed to a time-dependent field. This step is particularly relevant for the achievement of the final application herein envisioned, namely the use of MENPs for the combined action of nano-electroporation and controlled drug release. The analysis was divided into two phases, as sketched in Fig. 3: (1) for the reversible nano-electroporation phase, a constant *H*_DC_ of 300 mT (*i.e.*, the optimal value of *H*_threshold_^Rev-Nep^ obtained from the stationary parametric sweep study presented in paragraph 2.3) was applied and switched off after Δ*t*_Rev-Nep_ (time of nano-electroporation, arbitrarily chosen); (2) for the drug release phase, a sinusoidal wave was applied for Δ*t*_dr_ (time of drug release, arbitrarily chosen) with a frequency of 100 Hz and varying amplitudes (*H*_AC_ ranging from 1 mT to 6.5 mT). The first phase (at constant *H*_DC_) aimed at generating an electric field strong enough to induce reversible nano-electroporation on the cell membrane of a hypothetical cell located in the MENP nearby (within a distance of 5, 10 or 20 nm from the particle outer shell), while the subsequent sinusoidal phase facilitated the generation of alternating electric potentials on the surface of the nanoparticle, which is considered prodromal to drug molecule dissociation. The analysis was conducted for the two case scenarios presented in paragraph 2.1 (*i.e.*, culture medium and blood vessel walls).

To properly model the MENP behavior within a time-dependent study, the Jiles–Atherton (J–A) hysteresis model was implemented in the *Solid Mechanics* section in the software interface model builder, for the magnetostrictive core. The COMSOL entries in the core material settings for the J–A model parameters have been modified according to Table 4, considering our group's previous work.^27^ The mathematical equations governing the J–A model are summarized in the SI. In the *Study* section of the COMSOL interface, a *Time Dependent Study* was added, and a parametric sweep was imposed. The *Parameter Value List* section was filled in with specific values (such as 1, 2, 2.5, 3, 4.5, 5, and 6.5 mT). For the nano-electroporation phase, the magnetization profile was studied, paying particular attention to the value of magnetization obtained after Δ*t*_Rev-Nep_ (nano-electroporation time), *i.e.* when the constant static magnetic field was switched off. For the drug release phase, the core magnetization values, the electrical potential differences (Δ*V*) and the *α*_ME_, observed/calculated at a given time, were plotted in relation to the increasing amplitude of the sinusoidal wave. Ultimately, the concept of charge displacement was considered, derived as a function of the alternating magnetic field amplitudes and the efficiency factor (*α*_ME_). Such physical quantity, referred to as *Q*_ionic_, gives an indication of the stability involved in a hypothetical drug-MENP ionic bond (see the SI and Stimphil *et al.*^55^). All the outputs were processed *via* OriginPro 2023 (OriginLab, United States).

**Table 4:** Jiles–Atherton model parameters for magnetic hysteresis

Property	Symbol	Value	Reference
Magnetic saturation (A m^−1^)	*M*_S_	3.69 × 10^5^	48
Pinning loss (A m^−1^)	*k*	2 × 10^5^	56
Domain wall density (A m^−1^)	*a*	1.5 × 10^5^	56
Inter-coupling domain	*α*	1.5	56
Magnetic reversibility	*c*	0.3	56

## 3. Results

### 3.1 MENP baseline behavior under stationary field conditions

In the first part of the investigation (Fig. 1, step 1), a stationary study with a magnetic field above the material magnetic saturation (*M*_s_) was carried out to evaluate the magnetoelectric baseline behavior of the MENP in two separate environments. These stationary analyses aimed at validating (i) the generation of an electric potential at the surface of the particle and (ii) the establishment of an electric field in its vicinity, starting by studying the magnetization of all CFO domains, the following strain developed, and the subsequent stress generated at the BTO piezoelectric shell.

**Fig. 4:**
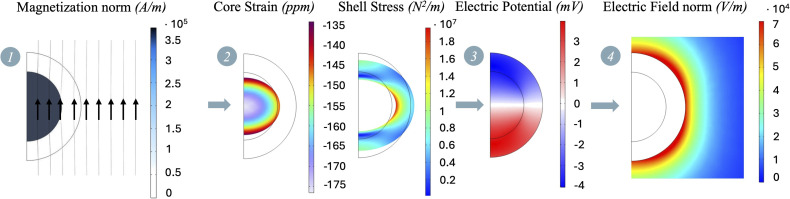
MENP baseline behavior under static stimulation conditions: from the magnetization of the CFO core (1), passing through the core strain and shell stress (2), to the generation of an electric potential on the particle surface (3), and the electric field in its vicinity (4).

The steps that a spherical magnetoelectric nanoparticle of our dimensions goes through, when ideally immersed in the blood vessel wall and exposed to a strong magnetic field of 2T (a field above its magnetization saturation, homogeneous and directed along the *z*-axis, considered the “easy axis” for the MENP magnetization) are shown in Fig. 4. Upon switching on the high intensity magnetic field, indicated by the black arrows pointing upwards, the CFO core becomes completely magnetized (Fig. 4 step 1, dark core, herein represented by the distribution of the magnetization, expressed in A m^−1^). Through deformation coupling of a magnetostrictive and piezoelectric material, magnetoelectricity can be exploited directly. Therefore, strain at the core level and stress at the shell level are reported (Fig. 4, step 2). The inhomogeneous stress distribution is the highest (but always within several tenths of ppm) along the sides of the nanoparticle. The magnetoelectric coupling causes a movement of charges, resulting in a maximum potential difference at a shell outer border of 6.07 mV (Fig. 4, step 3), and the consequent generation of an electric field in the MENP surrounding (Fig. 4, step 4), with values up to 7 × 10^4^ V m^−1^, sharply decreasing downwards with the distance from the outer surface of the shell. A similar electric behavior, with highly comparable values, is observed when the MENP is immersed in the culture medium, and the resulting distributions are shown in Fig. S2 and Table S1 of the SI.

### 3.2 Trend assessment of the MENP performance under stationary field conditions

**Fig. 5:**
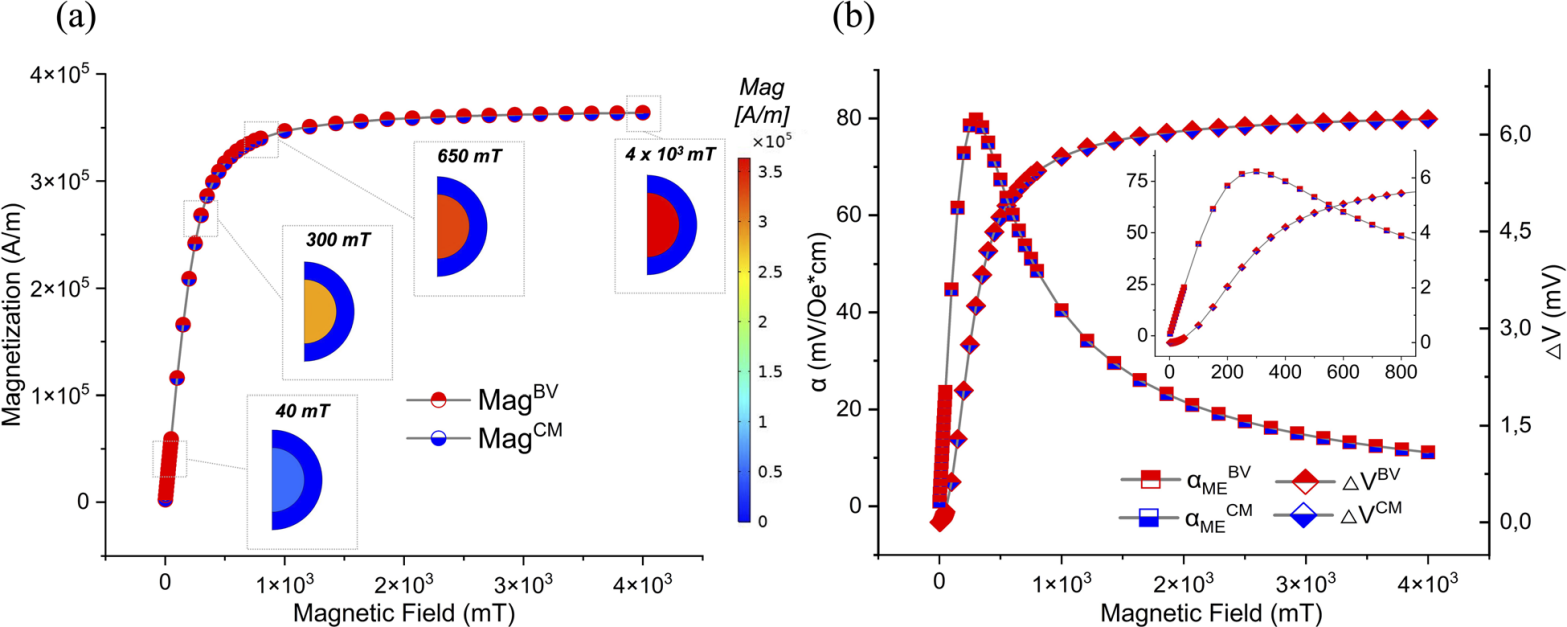
Magnetoelectric nanoparticle performance: (a) core magnetization trend of the MENP enclosed in the blood vessel wall (BV) or immersed in the culture medium (CM) at increasing applied magnetic fields (*H*_fields_); color-coded visualization is also included for the representative cases of *H*_field_ of 40 mT, 300 mT, 650 mT, and 4T. (b) Profiles of the magnetoelectric coefficient (*α*_ME_, expressed in mV Oe^−1^·cm^−1^) and the electric potential difference (Δ*V*, expressed in mV) developed across the MENP diameter, when the MENP is enclosed in the blood vessel wall (BV) or immersed in the culture medium (CM) at increasing applied *H*_fields_.

Subsequently, the magnetoelectric behavior of a single MENP in the presence of a wider range of static magnetic field values (from a low-intensity field of 2 mT to an intense field of 4 T) was investigated (Fig. 1, step 2). The magnetization profiles (magnetization, expressed in A m^−1^) registered at the core of the MENP for either the culture medium (in the following abbreviated as “CM”) case or the blood vessel wall (in the following abbreviated as “BV”) case, as a function of the applied external magnetic fields, are shown in Fig. 5a. In both environments, for low-intensity *H*_field_ (2–50 mT), the magnetization profile presents a linear trend, reaching a value of 4.72 × 10^4^ A m^−1^ (at 40 mT). Then, the linear tendency progressively translates into a curved profile (*H*_field_ of 100–800 mT and magnetization of 1.16–3.40 × 10^5^ A m^−1^), until it reaches a maximum magnetization value corresponding to an applied magnetic field of about 650 mT. Beyond this point, the magnetization remains constant, defining a plateau until the maximum magnetic field value applied in this study (4 T) is reached.

The MENP behaves as an electric dipole when exposed to an external magnetic field. To evaluate the magnetoelectric coefficient (*α*_ME_), according to eqn (1), the electric potential difference generated on the surface of the MENP was considered (Fig. 5b). The coefficient *α*_ME_ rapidly increases in the first part of the curve (*H*_field_ in the range of 2–50 mT), until it reaches a peak of about 79.82 mV Oe^−1^·cm^−1^, corresponding to a magnetic field of 300 mT. Beyond this value, the curve gradually decreases asymptotically, dropping down to 11.15 mV Oe^−1^·cm^−1^ at high-intensity *H*_field_ values. Fig. 5b also reports on the evolution of the electric potential (Δ*V*, calculated as the difference between the maximum and minimum values of the two opposite poles generated on the surface of the MENP, and expressed in mV), which shows a similar profile to that previously described for the magnetization: an initial linear trend at low-intensity *H*_field_ (reaching a value of 0.11 mV at 40 mT), followed by the attainment of a plateau (around 6.22 mV). Furthermore, the trend of this parameter in the two different environments under study appears to perfectly overlap.

### 3.3 Reversible nano-electroporation activation area

Next, the electric field generated by the particle in the different surroundings was analyzed. The external magnetic field (*H*_ext_) values considered are: *H*_ext(1)_, identified as 150 mT, corresponding to a value within the linear dependency between *α*_ME_ and *H*; *H*_ext(2)_, *i.e.* 300 mT, corresponding to the maximum *α*_ME_ (Fig. 5b), with the aim of taking advantage of the optimal performance of the MENP; and *H*_ext(3)_, namely 650 mT, corresponding to the magnetic field value at which the increase of two consecutive potential differences was below 0.15 mV (Fig. 5b). According to Fig. 1 (proceeding from step 2 towards step 3), the aim was to identify the optimal magnetic field (*H*_threshold_^Rev-Nep^) that could generate an electric field such as *E*_threshold_^Rev-Nep^ in the particle nearby.

**Fig. 6:**
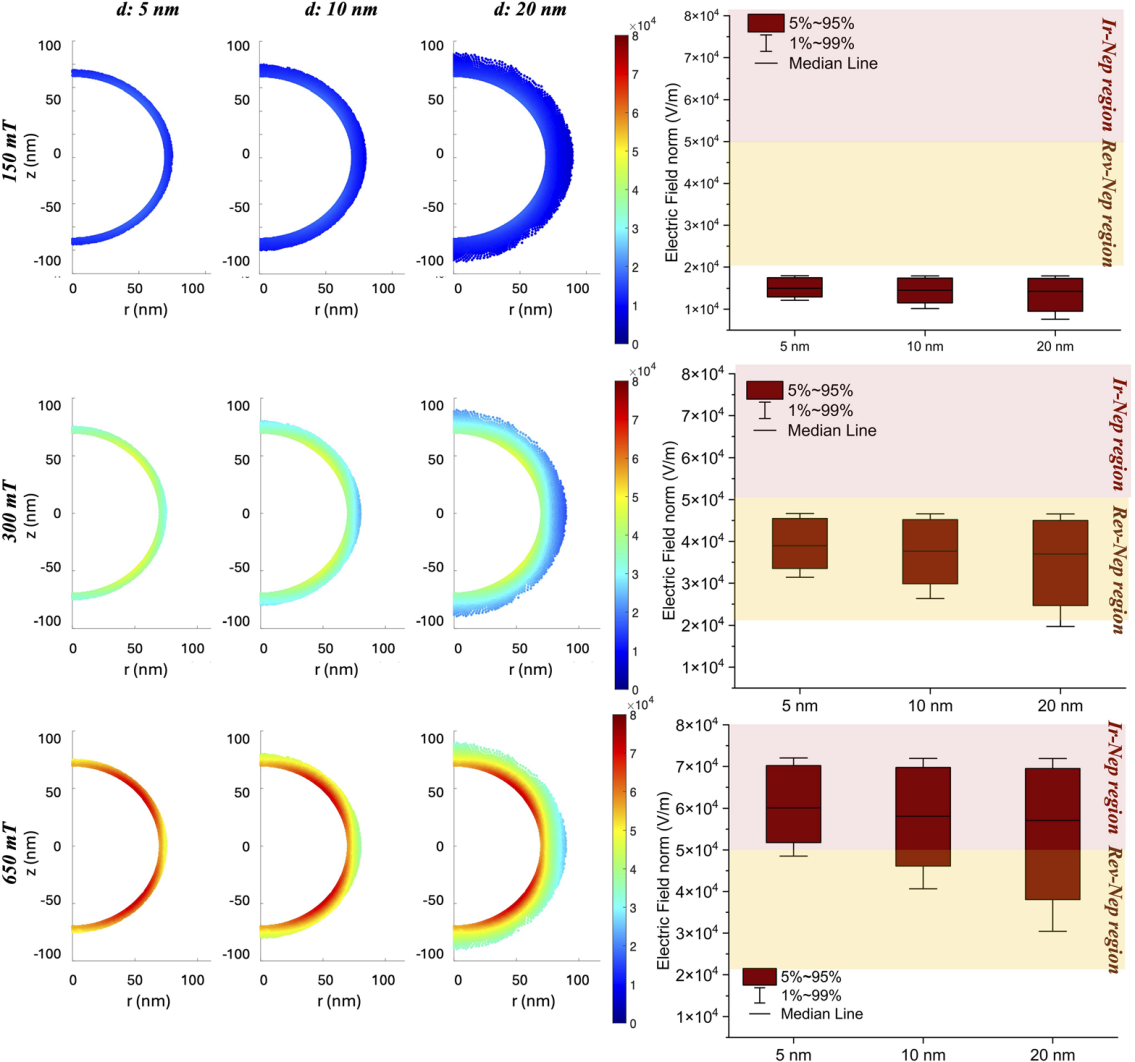
Nano-electroporation activation area. Left: distribution of *E*_field_ obtained after *H*_DC_ stimulation of a MENP engulfed in the blood vessel wall, at gradually increasing areas from the nanoparticle (*d* = 5 nm, 10 nm and 20 nm, distance from the outer shell); right: box plots of statistical metrics of *E*_field_ distributions in terms of median values, and the 1st and the 99th percentiles. Different nano-electroporation regions are highlighted: irreversible nano-electroporation (Ir-Nep region, in red) and reversible nano-electroporation (Rev-Nep region, in yellow).

The left side of Fig. 6 shows the electric field distributions at distances of 5, 10 and 20 nm from the outer border of the MENP shell (*r*_MENP_ = 70 nm), as extrapolated from COMSOL software and processed with MATLAB (see paragraph 2.2 in the Materials and methods section). On the right side of Fig. 6, the statistical metrics of the electric field distributions (in terms of median values and the 1^st^ and the 99^th^ percentiles) are reported, for each of the three progressively larger areas considered. Furthermore, the statistical metrics of the electric field distributions generated by the external magnetic fields under study are presented in Table 5, to ease the data comparison. While the data presented in Fig. 6 and Table 5 refer to the case of a MENP engulfed in the blood vessel wall, a similar analysis was also carried out for the case of the culture medium as the surrounding environment (Fig. S3 and Table S1). According to the data, stimulation with a magnetic field of 150 mT appears to be below the threshold that triggers a biological membrane response (above 2 × 10^4^ V m^−1^), while at 650 mT, *E*_fields_ distributions falling in the range of irreversible nano-electroporation are obtained (above 5 × 10^4^ V m^−1^). Therefore, maintaining conservative conditions, the following analyses have been carried out considering an external *H*_DC_ of 300 mT, whose generated *E*_fields_ distributions completely remain in the range of reversible nano-electroporation (2 × 10^4^ to 5 × 10^4^ V m^−1^).

**Table 5:** Statistical metrics in terms of median values and the 1^st^ and the 99^th^ percentiles of *E*_field_ distributions generated by *H*_ext(1)_, *H*_ext(2)_ and *H*_ext(3)_

	*H*_ext(1)_ = 150 mT			*H*_ext(3)_ = 300 mT			*H*_ext(3)_ = 650 mT		
(V m^−1^)	5 nm	10 nm	20 nm	5 nm	10 nm	20 nm	5 nm	10 nm	20 nm
Median	1.50 × 10^4^	1.45 × 10^4^	1.43 × 10^4^	3.90 × 10^4^	3.77 × 10^4^	3.70 × 10^4^	6.01 × 10^4^	5.81 × 10^4^	5.71 × 10^4^
99th percentile	1.80 × 10^4^	1.80 × 10^4^	1.79 × 10^4^	4.67 × 10^4^	4.66 × 10^4^	4.66 × 10^4^	7.21 × 10^4^	7.19 × 10^4^	7.19 × 10^4^
1st percentile	1.21 × 10^4^	1.02 × 10^4^	0.76 × 10^4^	3.14 × 10^4^	2.63 × 10^4^	1.97 × 10^4^	4.85 × 10^4^	4.06 × 10^4^	3.04 × 10^4^

### 3.4 Drug release operational ranges: time-dependent study

To complete our investigation (Fig. 1, step 3) and analyze the magnetoelectric behavior of the nanoparticle exposed to a time-dependent field, a time-dependent study was carried out. This step is crucial for achieving the final application herein envisaged, such as the exploitation of MENP-based nanotechnology for combined nano-electroporation and controlled drug release in cardiovascular applications.

**Fig. 7:**
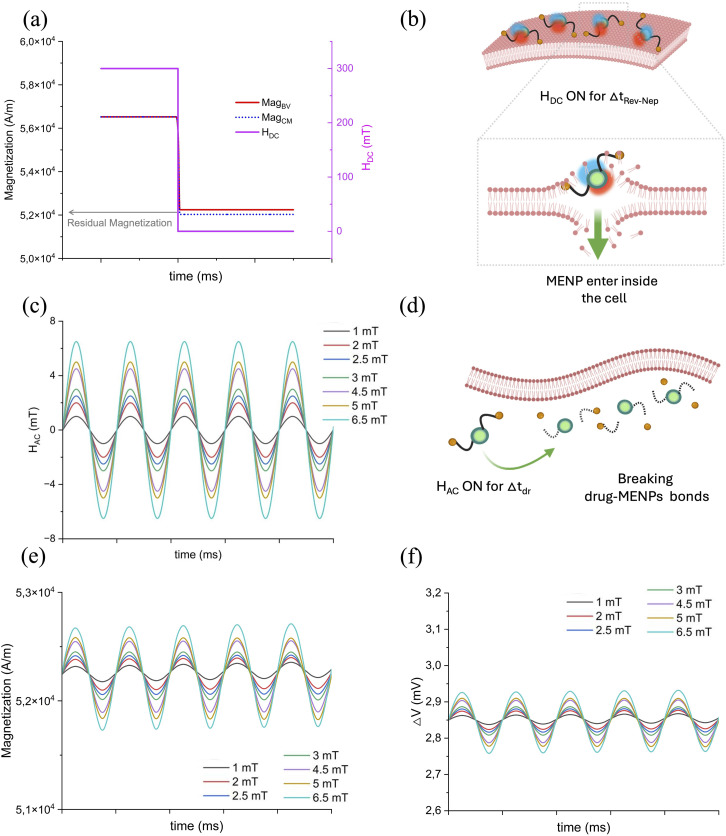
Time dependent study for drug release: (a) static magnetic field profile (*H*_DC_, expressed in mT), switched off after Δ*t*_Rev-Nep_, and core magnetization trend over time (magnetization, expressed in A m^−1^) in the blood vessel wall (red line) or in the culture medium (dotted blue line), to allow for a reversible cellular nano-electroporation; (b) schematic illustration of the hypothesized MENP journey to cross a cell membrane after application of a *H*_DC_ for the time required for the reversible nano-electroporation phenomenon to occur; (c) alternating magnetic field (*H*_AC_, expressed in mT) with variable amplitude profiles for Δ*t*_dr_ prodromal to the drug delivery process; (d) graphical representation of the hypothetical drug-MENP bond dissolution and drug release under *H*_AC_ stimulation; (e) MENP core magnetization behavior; and (f) electric potential difference profile over time for different *H*_AC_.

The analysis comprises two phases: (i) induction of reversible nano-electroporation on the cell membrane of a hypothetical cell in the vicinity of the MENP, triggered by a static magnetic field (*H*_DC_); (ii) promotion of the dissociation of drug molecules hypothetically bonded at the nanoparticle surface, by applying a *H*_AC_ stimulation, thus leveraging the generation of an alternating electric potential at the MENP outer shell. To achieve the reversible nano-electroporation, a *H*_DC_ of 300 mT (as obtained from the analysis reported in Fig. 6 and Table 1) is initially applied for Δ*t* = *t*_Rev-Nep_ (Fig. 7a, pink line) and then switched off. As this study is not intended to propose the definition of a precise protocol for nano-electroporation but is primarily interested in assessing the MENP-mediated process feasibility, specific time intervals are not considered. The magnetization profiles of the MENP core over time generated by such static magnetic field stimulation are reported in Fig. 7a for the case of both environments (blood vessel – red line, and culture medium – dashed blue line), with the aim of understanding how the nanoparticle core behaves during and after the removal of the stimulation. According to Fig. 7a, the core magnetization obtained at 300 mT is roughly 5.65 × 10^4^ A m^−1^ and remains constant over Δ*t*_Rev-Nep_. When the *H*_DC_ is switched off, the core retains a residual magnetization of approximately 5.2 × 10^4^ A m^−1^ (∼10 emu g^−1^) for both simulation environments, except for a negligible delta. Such residual magnetization is retained for *t* > *t*_Rev-Nep_, thus leaving the particle in a “magnetized” state.

Next, the drug release step is considered to investigate how the alternating magnetic field may affect the MENP specific behavior. In Fig. 7c, sinusoidal waves with the different amplitude values considered in the study are graphed. The core magnetization and the electrical potential difference profiles are plotted in Fig. 7e and in Fig. 7f, respectively, for a single MENP engulfed in the blood vessel well. For the case of the CM scenario, the corresponding plots and values are reported in SI, Fig. S4 and Table S2. As noticeable from Fig. 7e and f and Table 6, higher values of magnetization and Δ*V* are obtained when the amplitude of the sinusoidal stimulation is greater (*H*_AC_ ranging from 4.5 mT to 6 mT), with more pronounced oscillations, and values reaching 5.27 × 10^4^ A m^−1^ and 2.92 mV, respectively. Similarly to the previous analysis carried out for the trend assessment of MENP performance under steady state conditions (paragraph 3.2), the values of core magnetization, electrical potential difference and magnetoelectric coefficient related to the increase of the alternating field amplitude at a given time point (arbitrarily chosen) are reported in Table 6.

**Table 6:** MENP core magnetization, electric potential difference and magnetoelectric coefficient values in relation to the increase in the amplitude of the applied *H*_AC_ for the BV case. Each parameter is calculated at the instant when the peak of the *H*_AC_ sinusoidal wave is reached

*H*_AC_ (mT)	Magnetization (A m^−1^)	Δ*V* (mV)	*α*_ME_ (mV cm^−1^ Oe^−1^)
1	5.23 × 10^4^	2.86	87.36
2	5.24 × 10^4^	2.87	84.00
2.5	5.24 × 10^4^	2.88	83.43
3	5.24 × 10^4^	2.88	83.88
4.5	5.25 × 10^4^	2.90	81.70
5	5.26 × 10^4^	2.91	82.07
6.5	5.27 × 10^4^	2.92	79.85

**Fig. 8:**
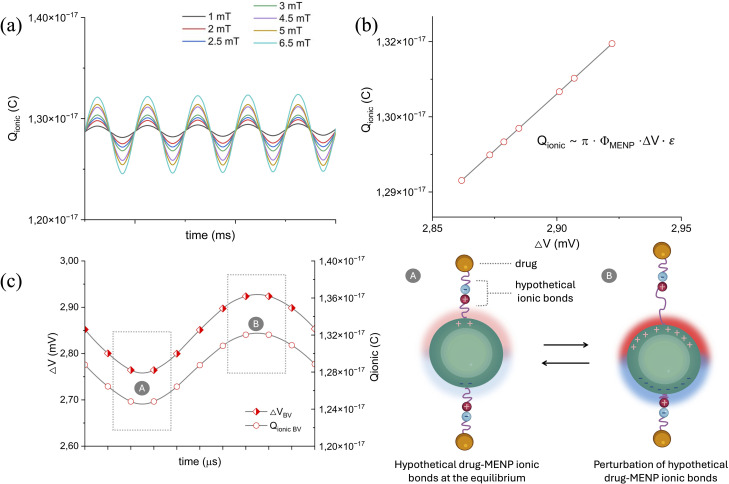
Ionic bond charge displacement parameter: (a) charge displacement in the hypothetical drug-MENP ionic bond profile over time for different *H*_AC_ amplitudes; (b) charge displacement in the hypothetical drug-MENP ionic bond values in relation to the various electric potential differences generated at the MENP surface at varying *H*_AC_ stimulations, considered at a specific time point; (c) comparison between the trends of the charge displacement in the hypothetical drug-MENP ionic bond and the electric potential difference over a certain period of time. In (c), as a representative example, the *H*_AC_ = 6.5 mT case was considered for the data presentation. Drug-MENP sketch: case A illustrates two hypothetical drug–MENP ionic bonds at equilibrium, where the electric potential difference is at its minimum and so the average displacement of charges is negligible; case B represents a perturbed state of the ionic bonds, characterized by the movement of the positive and negative charges from their equilibrium positions, which causes bond length stretching and bond length contraction. In A, less intense red and blue colorations indicate lower Δ*V* at the MENP surface, while in B more intense blue and red shadows refer to higher Δ*V*.

Fig. 8 reports simulation data comparison involving the concept of average charge displacement (referred to as *Q*_ionic_; the detailed explanation is available in the SI) to which hypothetical drug-MENP ionic bonds may be subjected when the system is immersed in a homogenous, low-intensity *H*_AC_ field at 100 Hz. The *Q*_ionic_ profiles at different *H*_AC_ amplitudes over the studied period reveal a trend comparable to that of the core magnetization and potential difference (Fig. 8a): an increase in the stimulation sine wave amplitude is associated with more visible oscillations. The susceptibility to rupture of hypothetical drug-MENP bonds is related to the alternating magnetic field values, which, in turn, cause oscillation of the electric potential difference. Therefore, the trend of *Q*_ionic_*versus* Δ*V* at a given time is also shown (Fig. 8b), highlighting a linear relationship, with charge shift values ranging from 1.29 to 1.32 × 10^−17^ coulomb (C), which are in line with Nair *et al.*^44^ Lastly, Fig. 8c compares the evolution over time of the electric potential difference and the average charge displacement of the hypothetical ionic drug-MENP bonds from their equilibrium positions. As a representative example, the *H*_AC_ = 6.5 mT stimulation case was considered for the BV scenario: as expected, *Q*_ionic_ and Δ*V* oscillate in a reciprocal manner. For the CM case, equivalent results are shown in the SI (Fig. S5).

## 4. Discussion

Nanotechnology advancements have recently brought to light new nanomaterials, which greatly facilitate the development of cutting-edge biomedical strategies to interact with biological systems and tissues in a wireless and on-demand way. Nanomaterials appear to be an intriguing option to face pathological problems in fields such as interventional cardiology and coronary artery surgery, due to their capacity to pass through biological barriers and allow a stimuli-responsive modulation of the anti-inflammatory or anti-proliferative drug release. Notably, magnetoelectric (ME) nanomaterials, able to induce electrical polarization on their surface when exposed to a magnetic field, constitute a one-of-a-kind tool, whose properties and stimuli-responses can be remotely activated and tuned by acting on their material composition, size, geometrical anisotropy, and combination with organic compounds.^57,58^ Among the most widely studied ME material configurations are core–shell magnetoelectric nanoparticles (MENPs), whose structure allows the maximization of interfacial coupling between the magnetostrictive core phase and the piezoelectric shell phase, thus optimizing the performance.^27,54^ The most widely used and promising material combination is based on a cobalt ferrite (CFO) core and a barium titanate (BTO) shell, presenting good biocompatibility and a stable magnetoelectric effect.^59^ A quantification of the MENP performance is given by the magnetoelectric coefficient (*α*_ME_), which considers the magnetoelectric coupling in terms of the generated electric field (output) and the external magnetic field (input). There are a limited number of studies in the literature reporting or mapping *α*_ME,_ due to difficulties in measuring it experimentally and the lack of standardized protocols (*e.g.*, single particle *vs.* bulk materials, in-house measurement apparatus). Such a limitation is related to the intrinsic material parameters of the components, which, in turn, are influenced by complex structural features such as magneto-crystalline anisotropy, domain distribution, domain wall motion of the phases, crystal lattice mismatch and the presence of point defects at the interface.^28^ Computational analyses come to the rescue by deriving significant parameters and predicting the overall behavior of the system. In this regard, recent simulation studies have reported values of *α*_ME_ ranging from 0.01 V cm^−1^·Oe^−1^ to approximately 2.5 V cm^−1^·Oe^−1^, to define the MENP conversion efficiency under various combinations of static and alternating magnetic fields and by examining various shapes and geometries.^27,28^

The *in silico* work starts with a validation of the ME effect of a MENP engulfed in the blood vessel wall or immersed in the culture medium, as modeling environments, to observe the generation of an electric potential on the MENP surface and an electric field in the surroundings, in response to a 2T static magnetic field (above the core magnetization saturation). The MENP baseline behavior is analyzed in Fig. 4: the magnetization involves a realignment of the magnetic domains within the core, a compression along the direction of the applied magnetic field (*i.e.*, the *z*-axis), and an expansion in the other two dimensions, leading to a core dimensional change (lattice deformation) on the order of a hundred ppm. This deformation is imparted to the piezoelectric shell, transforming the stress exerted by the core into a change in the charge position within the atoms of the piezoelectric lattice, thus generating a surface potential and an electric field distribution on the MENP and in its vicinity, which are consistent with those found in previous studies conducted by our group,^30^ where MENPs have been proposed for peripheral nerve stimulation^29,60^ and sensing,^61^ and nerve regeneration,^62^ highlighting the versatility of the adopted computational framework. After having confirmed the basic response of the MENP, a wider range of DC magnetic field values is assessed, and the CFO-BTO nanoparticle parameters are thoroughly characterized. The results of the calculated magnetization recorded at the core of the MENP (Fig. 5a) describe a curve whose trend agrees with the one of Ma *et al.*^63^ In fact, when the applied magnetic field gradually increases, the domains become progressively aligned with its direction and the magnetization values rise. At a certain point, the magnetization profile reaches a plateau, because of the complete orientation of the magnetic domains in the direction of the applied field, and any further increase in the external stimulus does not translate into a stronger magnetization. The trend of the magnetoelectric coefficient as a function of the applied field (Fig. 5b) is in accordance with the work of Kumari *et al.*^28^ Considering that MENPs of different sizes and material properties are modelled, our maximum value of *α*_ME_ is about 0.1 V Oe^−1^·cm^−1^, registered at 300 mT, while they reported a peak of 0.4 V Oe^−1^·cm^−1^ corresponding to an external *H*_DC_ of 600 mT. It is therefore possible to conclude that the MENP performance does not necessarily improve as the potential difference increases, but it rather diminishes at the expense of a progressively higher magnetic field.

By tuning the magnetoelectric coupling, a wide range of diagnostic and therapeutic applications based on MENPs can be explored. In this context, ME properties can be exploited to wirelessly and selectively manipulate cells and even reversibly permeabilize their membrane to accomplish targeted drug delivery. Efficient permeabilization of the membrane is conventionally achieved by following protocols based on the use of electric fields (*i.e.* electroporation), which generally rely on bulk electrodes placed in the proximity of cells and/or tissues. Plate electrodes for non-invasive stimulation or needle electrodes inserted into the tissue for invasive stimulation are two types of traditional devices.^39,40^ Invasive stimulation is usually the most effective strategy for electroporation, but it suffers from drawbacks common to all invasive procedures (complications can include bleeding, inflammatory responses and infections or systemic issues). These latter hinder the use of such a technique for broad-spectrum treatments or when the localization of the diseased area might be difficult to reach (*e.g.*, cardiovascular applications). Recently, some studies have focused on the use of inorganic nanoparticles, such as gold nanoparticles or MENPs, as a completely contactless method to achieve electroporation with enhanced spatial specificity at the nanoscale.^42,64^ In particular, in the very recent work of Bryant *et al.*,^65^ MENPs have been explored as nanotheranostic agents for specific targeting of cancer cells and damaging *via* irreversible electroporation (IRE) by means of a high amplitude DC magnetic field (on the order of magnitude of 7T). From the simulations carried out in our study (Fig. 6 and Table 5), the electric field values considered suitable for reversible nano-electroporation (*i.e.*, ranging from 2 × 10^4^ to 5 × 10^4^ V m^−1^, according to Lv *et al.*^41^ which we have taken as reference despite referring to bulky electrode-mediated pulsed stimulation) are reached when a *H*_DC_ of 300 mT is used to excite the MENP. This corresponds to the optimal performance scenario where the maximum efficiency (*α*_ME_) is observed. In contrast, when the induced electric field exceeds 5 × 10^4^ V m^−1^ (corresponding to a *H*_DC_ of 650 mT), cell death caused by irreversible nano-electroporation has been ascribed to several reasons,^66^ such as permanent membrane lysis, loss of cell homeostasis, flush in/out of molecules, and even cytotoxic effects as a consequence of electrolysis and by-products generated around the electrodes.^66^

Having verified the occurrence of reversible nano-electroporation in the presence of an external 300 mT *H*_DC_, we proceeded with a time-dependent static simulation. The MENP, which exhibits its non-superparamagnetic properties at room temperature,^24^ presents a residual magnetic flux density^48^ when a *H*_DC_ of 300 mT is applied for Δ*t* = *t*_Rev-Nep_ and subsequently removed (Fig. 7a). To further investigate the potential of MENPs not only as wireless permeabilizing nanodevices of biological membranes but also as emerging tools for the controlled release of therapeutic agents into specific tissues, we focused our investigation on the alternating magnetic field simulation. Ideally, it has been previously postulated that the application of a *H*_AC_ onto a magnetized nanoparticle decorated with drug molecules would create conditions for an efficient drug-MENP bond breaking process, thus enhancing the drug release efficacy.^44^ Such phenomenon is influenced by several factors, either related to the physico-chemical features of the system (*i.e.*, organic coating on the MENPs, nature of the drug-MENP bond, and type and amount of drug loaded), or the conditions of magnetic stimulation (duration, amplitude and frequency of *H*_AC_ and possible temperature increase during the stimulation).^43,67^ In this preliminary computational study, the main parameter considered was the variation of the *H*_AC_ amplitudes (values in the range of the experimental published literature^43,67^), from which the core magnetization and the subsequent electric potential difference at the MENP surface are dependent. In line with the work of Kozielski *et al.*,^68^ where it is affirmed that applying a sinusoidal magnetic field to magnetoelectric materials produces a sinusoidal electric field with a frequency that matches the input, Fig. 7e and f display comparable trends for the core magnetization and the electric potential difference profiles. As a consequence of the ME effect, the electric dipole generated at the MENP outer border induces a charge surface density (defined as *σ*_MENP_) of the order of magnitude of ±*αH* (following the theoretical dissertation of Stimphil *et al.*,^55^ eqn (18) of the SI). Assuming a hypothetical drug molecule attached at the MENP outer shell *via* an ionic bond, its release would occur if such *σ*_MENP_ equals and/or exceeds the charge density involved in the drug-MENP ionic bond. Moreover, fundamental to the dissolution of such a hypothetical ionic bond is the effective alternation of stretching and contracting of the ionic bond length, which ultimately results from the movement of positive and negative charges that constitute the ionic bond itself from their equilibrium positions. Considering that multiple drug molecules may be bound to a single MENP surface, an average positive/negative charge displacement can be reasonably defined (labelled as *Q*_ionic_, according to the theoretical modelling of nanoparticle-bound drug release by Stimphil *et al.*,^55^ eqn (19)–(22) of the SI). Our simulations conducted under varying *H*_AC_ conditions (Fig. 8b) highlight that such an average charge displacement in the hypothetical ionic bond is indeed governed by a linear dependence on the electrical potential difference (Δ*V*) developed at the MENP surface. Δ*V* oscillations over time would promote *Q*_ionic_ variations, thus perturbing the equilibrium positions of the positive and negative charges. We speculate that the length of the hypothetical drug-MENP bonds would be far from their equilibrium when such oscillations become more readily detectable (*i.e.*, *H*_AC_ equal to or greater of 4.5 mT, Fig. 8c). Higher alternating magnetic field values would result in larger oscillation amplitudes, thereby spanning over a wider range of electrical potential differences at the MENP outer shell, which, in turn, would be responsible for a stronger variation in the average bond charge displacement. In other terms, such stimulation conditions would cause a strong ‘shaking effect’ on the system, favoring the delivery of the therapeutic agent.^43^

### 4.1 Study limitations

To the best of our knowledge this work marks the first *in silico* assessment of the operational parameters (*i.e.*, *H*_DC_ and *H*_AC_ external stimulations, with varying field strengths and amplitudes) involved in MENP-mediated nano-electroporation and drug release. However, several factors that are known to contribute to both phenomena are not included in the herein computational model and data analysis. While our study focused on the MENP general electrical outputs (*i.e.*, *E*_fields_ in the MENP nearby and Δ*V* at its surface after remote activation *via* external magnetic fields) and how they set the conditions for MENP-biological structure interface events to occur, other aspects are neglected. In fact, our model is an approximate simplification of the MENP structure, that does not consider additional outer layers, such as organic coating linkers and drug molecules, which are expected to slightly modify the overall electrical behavior. Regarding the nano-electroporation process, membrane pore dynamics formation has not been considered at this stage of the investigation and any possible effect of the thermal contribution related to MENP stimulation is herein overlooked. In providing a high-level physical perspective on the feasibility of using DC electric fields generated by MENPs, the concepts of free moving charges in conductive media and Debye length have not been considered in the present work. More detailed investigations will be addressed in future studies to investigate the electric phenomena at the MENP–cell membrane interface, taking into account the screening effects of free charge carriers on the expected cross membrane electric potential generated by the MENPs located within the Debye length. Such a limitation is also related to the computational challenge of modeling systems with highly dissimilar dimensions and spatial occupation, ranging from the nanometer sized features of the MENPs to hundreds of micrometers of extension of cell membranes. Lastly, future studies more specifically centered on the modeling of the MENP–therapeutic agent bond are needed, to correlate both the DC and AC magnetic stimulation with the drug delivery process in terms of bond nature, the amount of drug loaded, and the inorganic particle/organic molecule assembly stability.

## 5. Conclusion

Today, MENPs are at the forefront in the development of innovative, wirelessly powered biomedical devices to actively interact with cells and tissues. Based on a spherical MENP model previously validated by our group, the current work aims to explore the use of MENPs for reversible nano-electroporation and drug release. The first part of our step-by-step analysis showed how to best exploit the operating principle – maximizing *α*_ME_ – so that the induced electric field in the MENP nearby, following static external magnetic field activation, is within the range of a reversible nano-electroporation, not harmful for the cells. The second part focused on the analysis of the drug release phenomenon, shedding some light on the reciprocal dependence of the magnetoelectric effect and the subsequent changes in the electric potential on the charge displacement. The preliminary concept herein presented constitutes a precursor to detailed analyses of gradually more complex systems, which will take into account the effects of organic coatings and multiple drug molecules on the overall MENP electrical behavior and on the charge displacement at the MENP–molecule interface. This will provide further knowledge to support the synthesis of drug-loaded MENPs and to define protocols for specific delivery systems adapted to cardiovascular surgery applications, in view of a progressively more personalized and less invasive medicine.

## Author contributions

AT: investigation, visualization, data curation, writing – original draft; GS: conceptualization, visualization, and writing – original draft; SF, EC, and MP: data curation and methodology. SF, EC, MP, ML, AM, and BC: writing – review and editing; PR and EC: funding acquisition. MP, BC and PR: supervision. All authors contributed to manuscript revision and read and approved the submitted version.

## Conflicts of interest

The authors declare no conflict of interest.

## Supplementary Material

NA-OLF-D5NA00438A-s001

NA-OLF-D5NA00438A-s002

NA-OLF-D5NA00438A-s003

NA-OLF-D5NA00438A-s004

NA-OLF-D5NA00438A-s005

NA-OLF-D5NA00438A-s006

NA-OLF-D5NA00438A-s007

## Data Availability

The data supporting this article have been included as part of the SI. Data for this article, including material properties are taken from the COMSOL Multiphysics Library, available at https://www.comsol.com, while the tissue properties are taken from the IT’IS Foundation Tissue Database of the Sim4Life database, available at https://itis.swiss/virtual-population/tissue-properties/database/dielectric-properties/. Supplementary information include the mathematical expressions for (a) the COMSOL multiphysics model of the magnetoelectric phenomenon; (b) the COMSOL multiphysics model of the magnetic hysteresis phenomenon; (c) the theoretical model of the drug delivery phenomenon; (d) magnetization loop of the cobalt ferrite core; (e) static study results to validate the MENP baseline behavior in the culture medium environment; (f) distributions of the electric field produced by various external *H*_DC_ fields within culture medium surroundings; (g) time dependent study for the drug release step in the CM environment; (h) MENP core magnetization, electric potential difference and magnetoelectric coefficient values in relation to the increase in amplitude of the applied *H*_AC_ for the CM case scenario. See DOI: https://doi.org/10.1039/d5na00438a.
