# Supplementary figures and images for: Exploring magnetoelectric nanoparticles for advanced nano-electroporation and drug delivery in interventional cardiology
Source: Nanoscale Adv. 2025 Aug 22;7(19):5978–92. doi: 10.1039/d5na00438a (PMC12434618; doi:10.1039/d5na00438a)

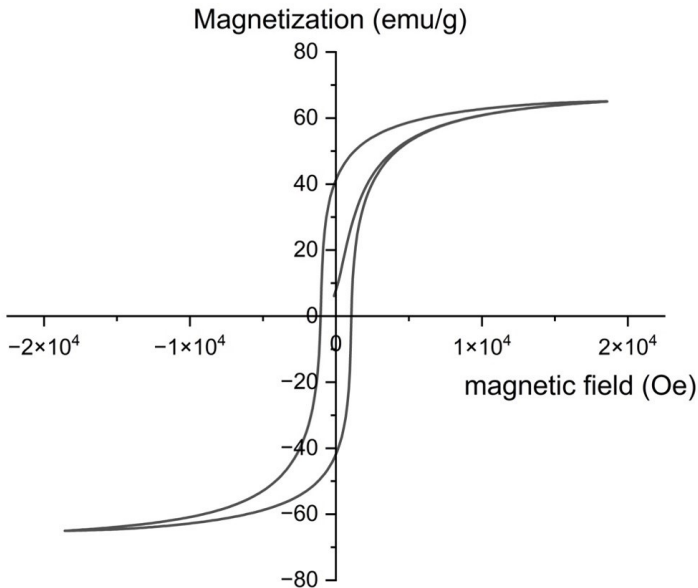

Supplement: NA-007-D5NA00438A-s002 [file NA-007-D5NA00438A-s002.pdf]

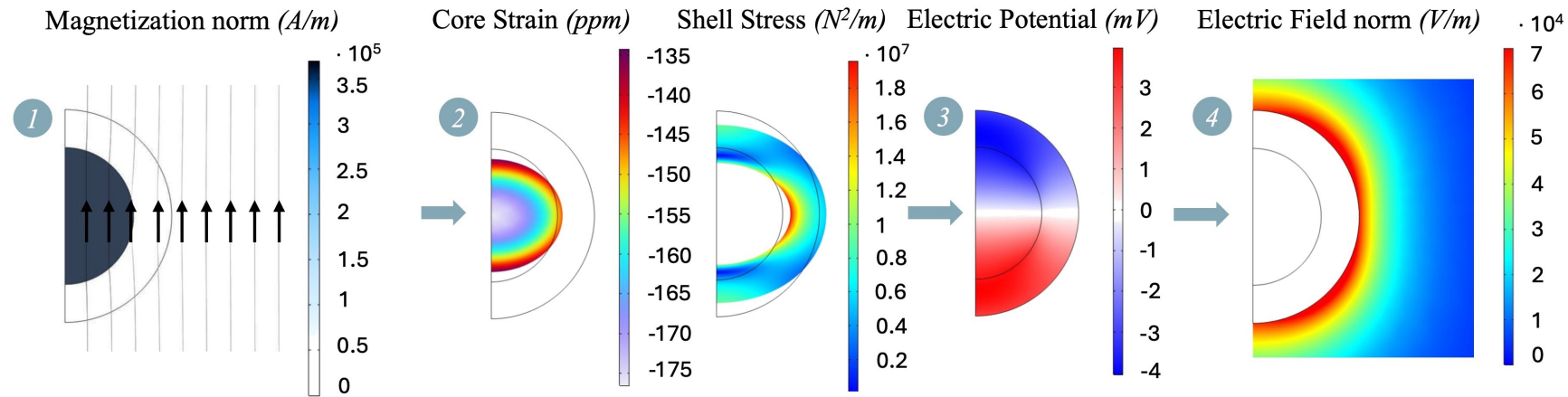

Supplement: NA-007-D5NA00438A-s003 [file NA-007-D5NA00438A-s003.pdf]

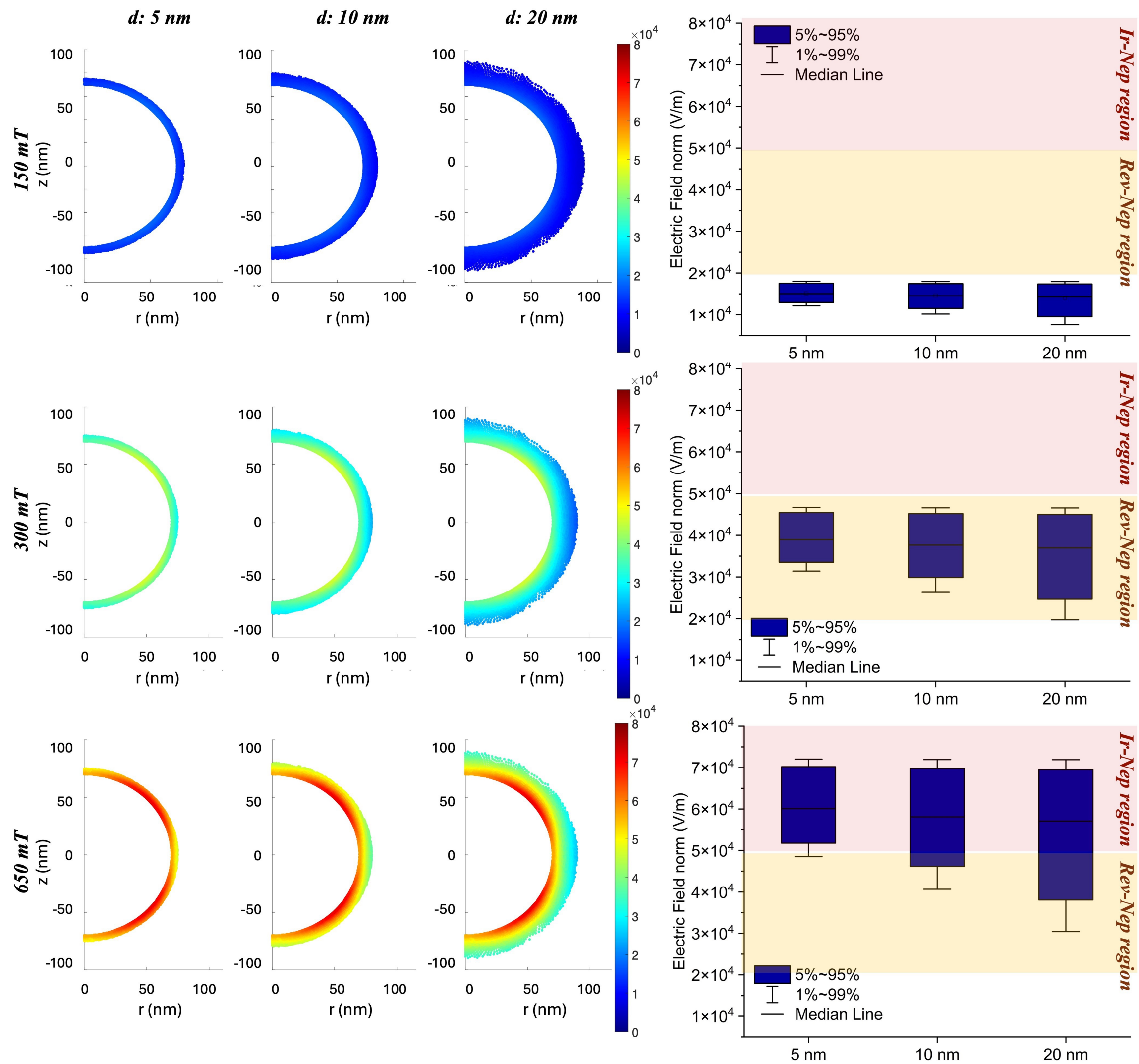

Supplement: NA-007-D5NA00438A-s004 [file NA-007-D5NA00438A-s004.pdf]

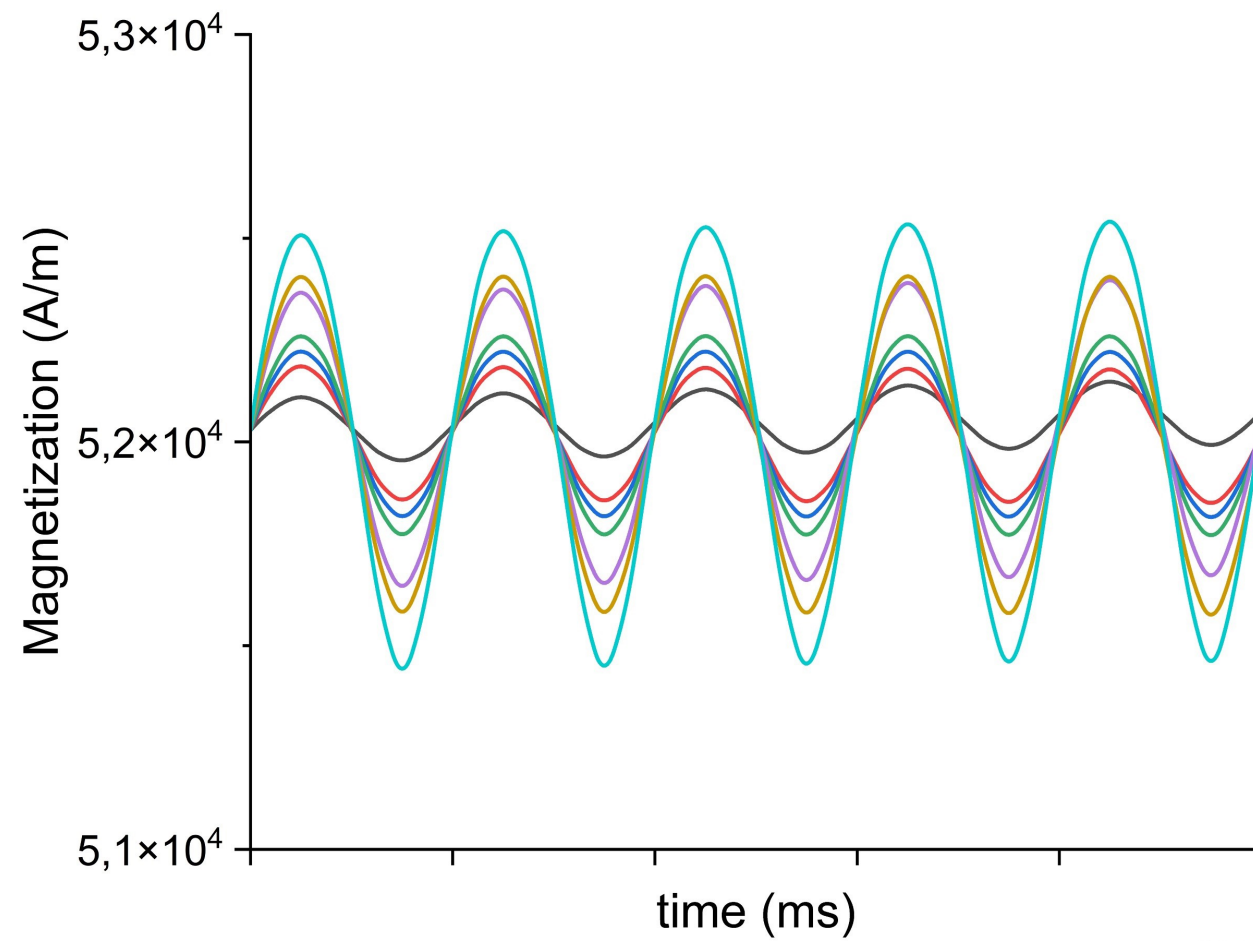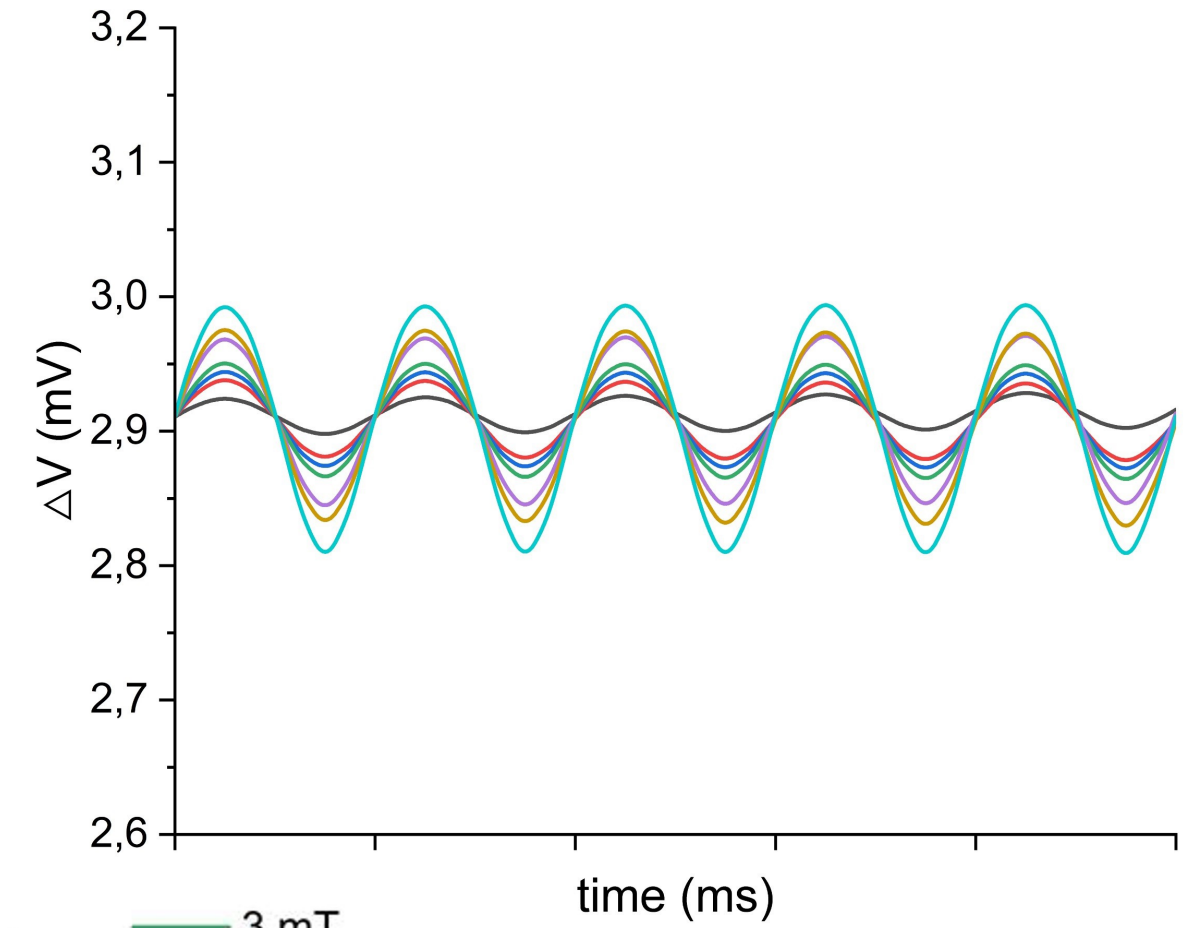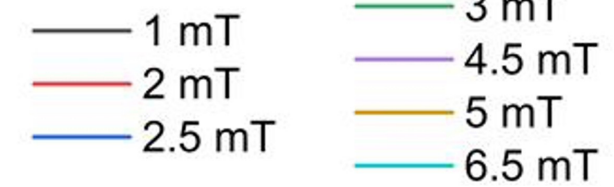

Supplement: NA-007-D5NA00438A-s005 [file NA-007-D5NA00438A-s005.pdf]

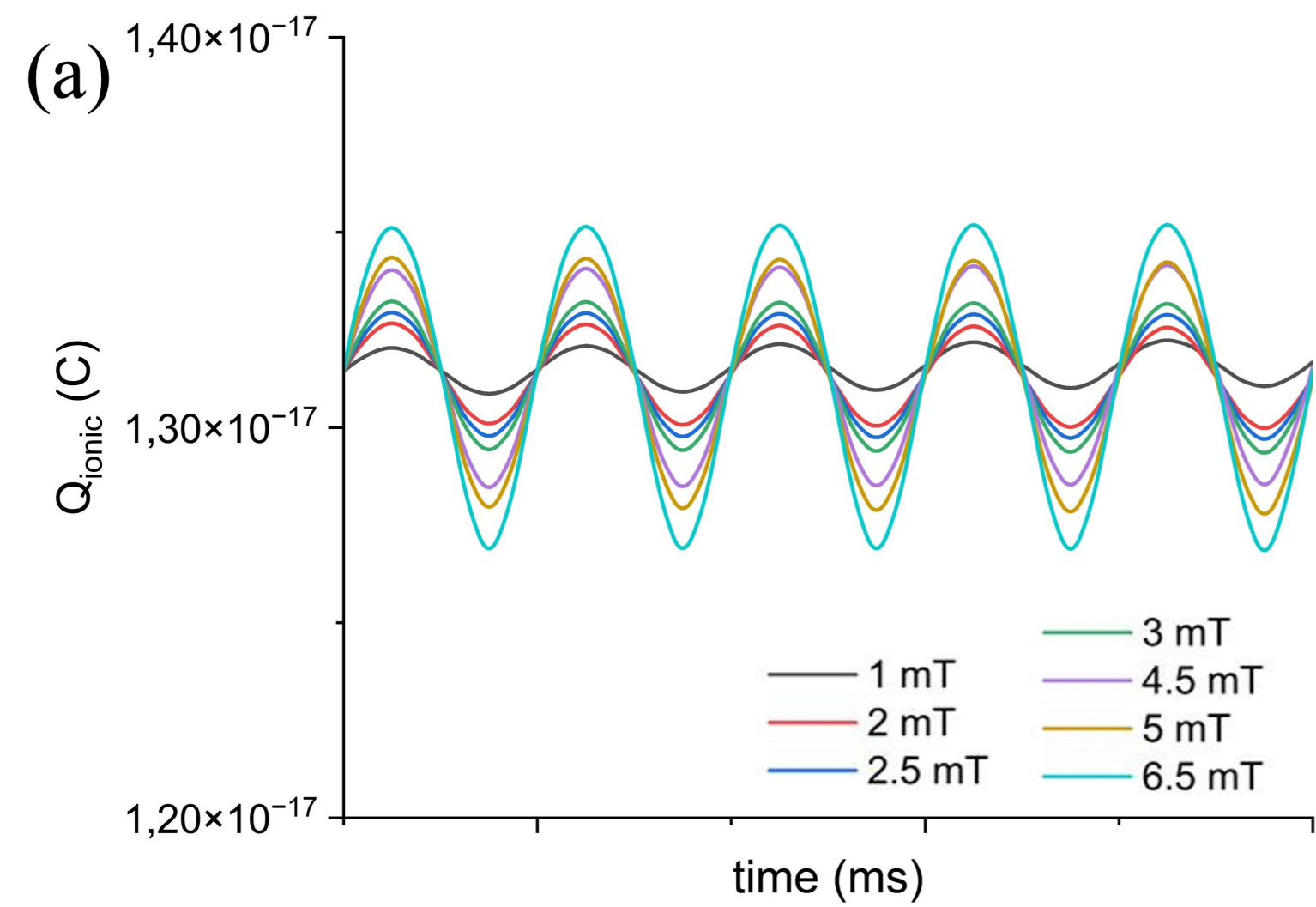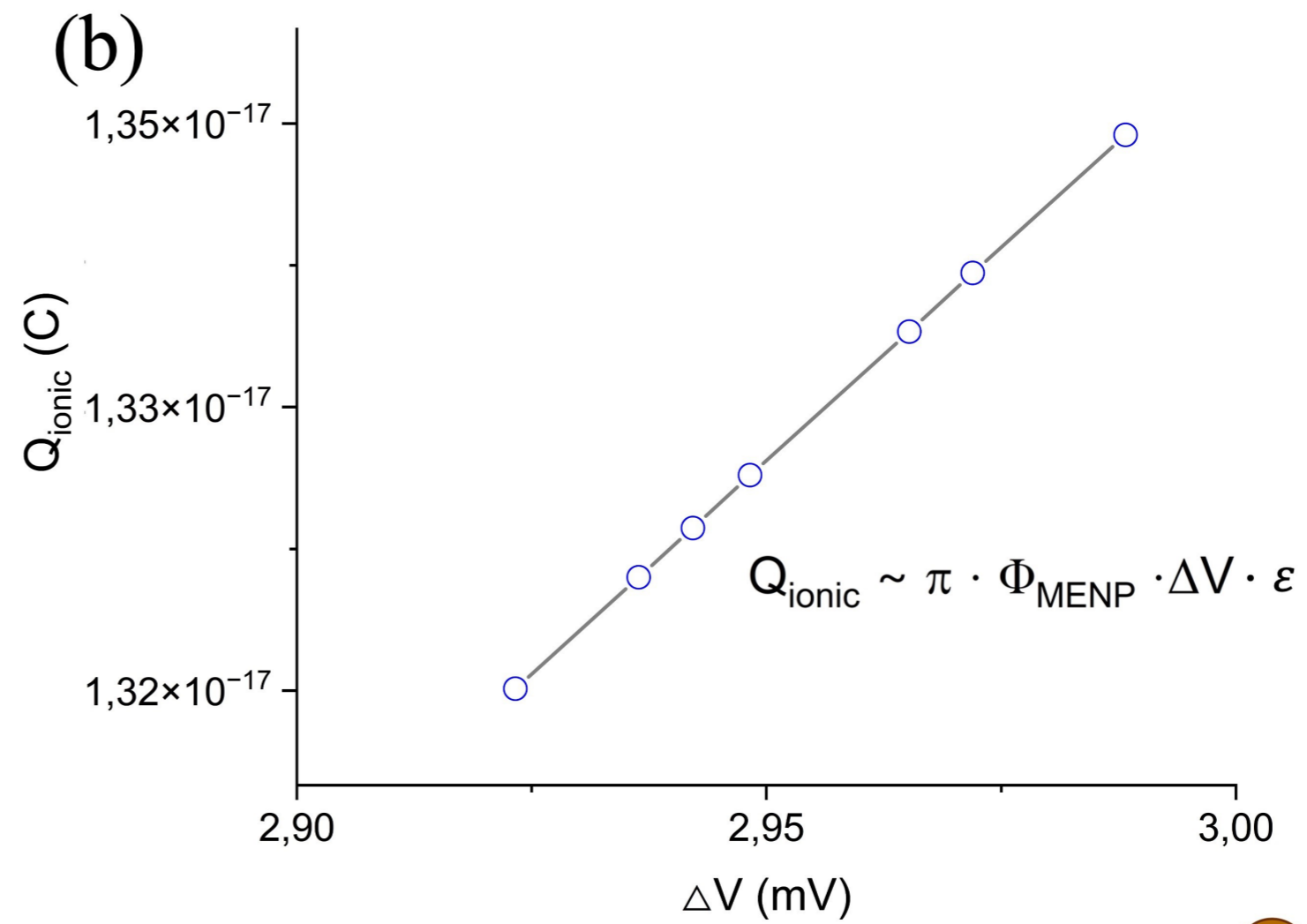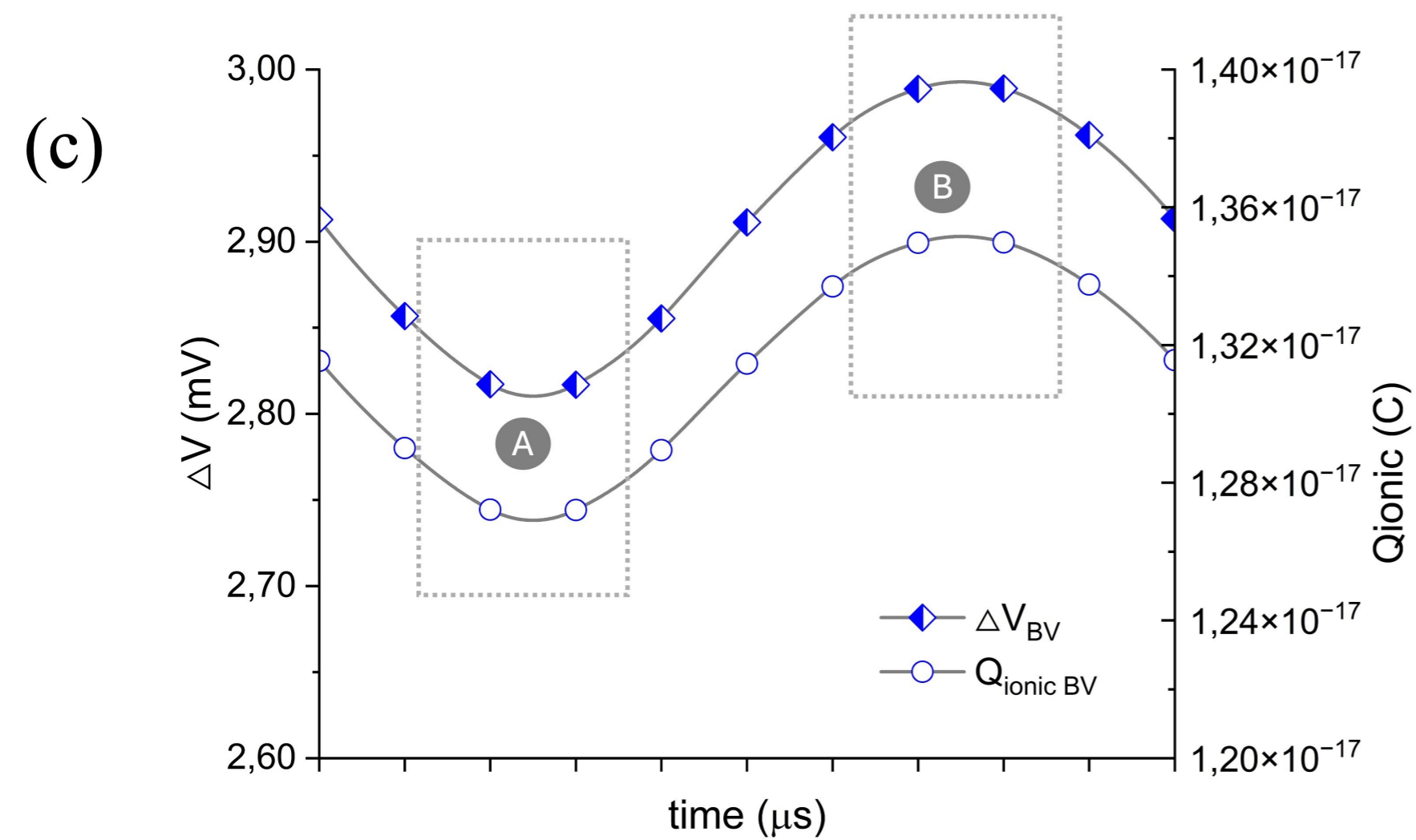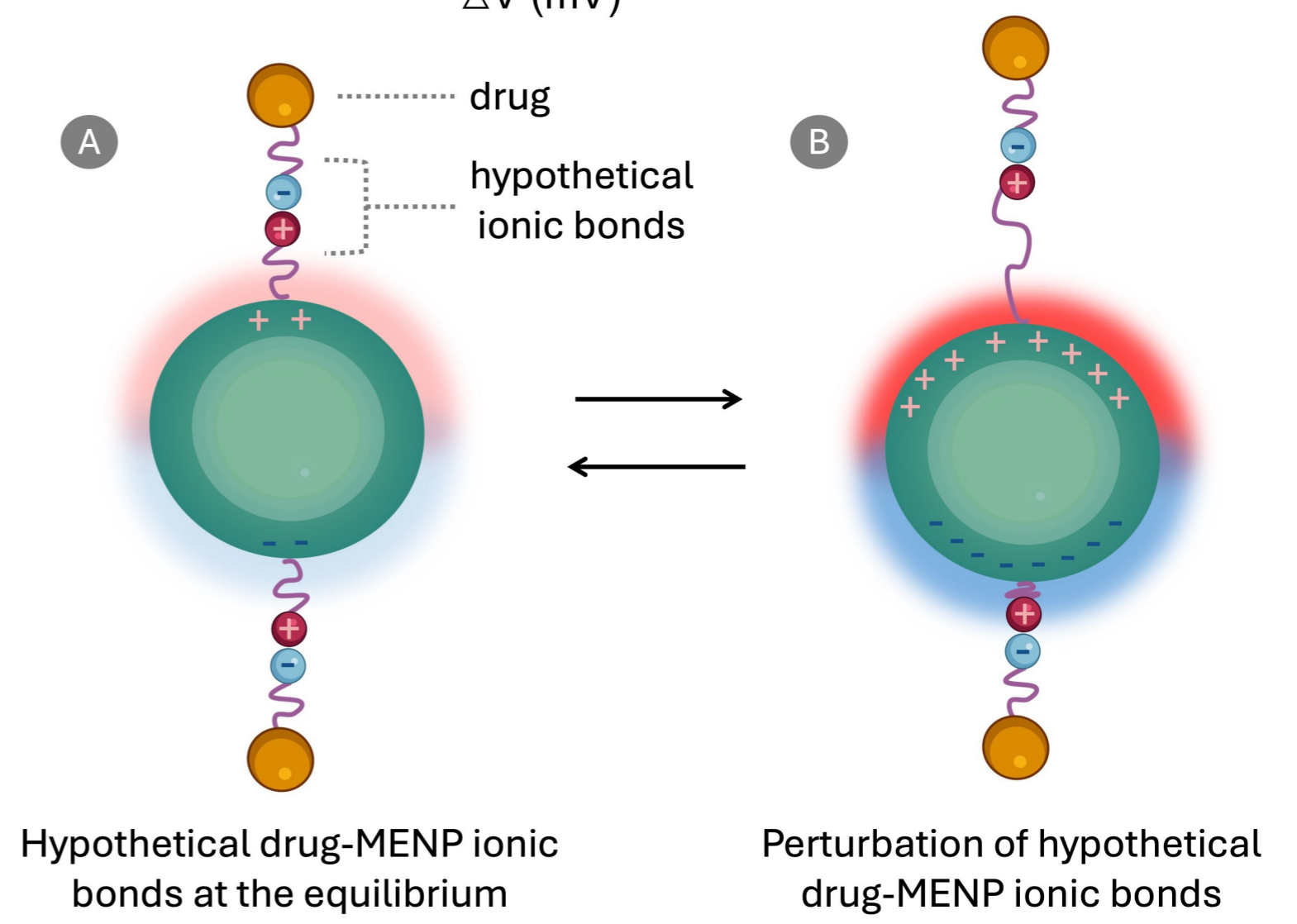

Supplement: NA-007-D5NA00438A-s006 [file NA-007-D5NA00438A-s006.pdf]
