# Supplementary material for: Exploring magnetoelectric nanoparticles for advanced nano-electroporation and drug delivery in interventional cardiology
Source: Nanoscale Adv. 2025 Aug 22;7(19):5978–92. doi: 10.1039/d5na00438a (PMC12434618; doi:10.1039/d5na00438a)
Supplement: NA-007-D5NA00438A-s007 [file NA-007-D5NA00438A-s007.pdf]

Supplementary Information (SI) for Nanoscale Advances.
This journal is © The Royal Society of Chemistry 2025

Exploring magnetoelectric nanoparticles for advanced nano-electroporation and drug delivery in interventional cardiology

A. Tommasini[1,2], G. Suarato[2*], S. Fiocchi[2], E. Chiaramello[2], A. Marrella[2], M. Lenzuni[2], M. Parazzini[2], B. Cortese[3,4,5], P. Ravazzani[2]

[1] *Dipartimento di Elettronica, Informazione e Bioingegneria, Politecnico di Milano, Piazza Leonardo da Vinci 32, 20133, Milano, Italy*
[2] *Cnr-Istituto di Elettronica e di Ingegneria dell'Informazione e delle Telecomunicazioni, Piazza Leonardo da Vinci 32, 20133, Milano, Italy*
[3] *Interventional Coronary Center, University Hospitals Harrington Heart & Vascular Institute, Cleveland, Ohio, United States*
[4] *Fondazione Ricerca e Innovazione Cardiovascolare, Via E. Ponti, 49, 20136, Milan, Italy*
[5] DCB Academy, Via E. Ponti, 49, 20136, Milan, Italy

*Corresponding Author: giulia.suarato@cnr.it

Supplementary Materials

COMSOL Multiphysics model of the Magnetoelectric Phenomenon

The mathematical equations governing the magnetoelectric coupled model have been thoroughly described by Fiocchi *et al.*[1] and summarized below.

(A) Magnetic Field Module

In the Magnetostatics mode, the relationship between the magnetic flux density (**B**) and the magnetic field (**H**) can be expressed through the following equation:

$$B = \mu_0 \mu_r H \tag{1}$$

This equation applies to the piezoelectric shell and the surrounding non-magnetic regions. For the magnetostrictive core, the nonlinear relationship between the magnetization (**M**) and the magnetic field (**H**) is given by:

$$B = f(|H|) e_H \tag{2}$$

where $f(|H|)$ is a function of the magnitude of **H** and $\mathbf{e_H}$ is the unit vector in the direction of the magnetic field **H**.

(B) Solid Mechanics Module

Different materials features (e.g., Young's modulus, Poisson's ratios, densities, etc.,) are involved in the computational solid mechanics module. The magnetostrictive cobalt ferrite core is governed by equation (3), which represents the relation between the magnetostrictive strain (ε_{ME}) of an isotropic material with its magnetic properties:

$$\varepsilon_{ME} = \frac{3\lambda_s}{\mu_0 M_s^2} dev(M_s \otimes M) \tag{3}$$

In this equation, λ_s is the saturation magnetostriction (ppm), μ_0 defines the relative permeability, M_s is the magnetic saturation (A/m) and M refers to the total magnetization.

For what concerns the mechanical stress at the piezoelectric shell, equation (4) applies:

$$S = S_0 + C : \varepsilon + E \cdot e \tag{4}$$

where e is the piezoelectric Voigt coupling matrix representing the stress tensor, C is the elastic right Cauchy deformation tensor, and E is the electric field computed by using the piezoelectric coupling.

(C) Electrostatic Module

In the Electrostatic module, Gauss' Law is solved:

$$\nabla \cdot D = \rho_v \tag{5}$$

$$E = -\nabla V \tag{6}$$

where D is the electric flux density and ρ_v is the volume charge density.
More specifically, in the piezoelectric shell the relation between the electric flux density D and the solid mechanics variables is given by equation (7):

$$D = \varepsilon_0 E + \varepsilon_0 \chi E + e : \varepsilon \tag{7}$$

where ε_0 is the vacuum electric permittivity and χ is the relative electrical susceptibility.

COMSOL Multiphysics Model of the Magnetic Hysteresis Phenomenon

The mathematical equations for the Jiles-Atherton model have been previously described by Marrella *et al.*[2] and reported below.

The main equation in the Jiles-Atherton model describes the variations in the total magnetization **M** due to the changes of the effective magnetic field as follows:

$$\frac{dM}{dt} = max(\chi\, dHeff,0)\frac{\chi}{|\chi|} + c\frac{dM_{an}}{dt} \tag{8}$$

Where c is a measure of the magnetization reversibility and the auxiliary vector χ is defined as:

$$\chi = \frac{M_{an} - M}{k} \tag{9}$$

with M_{an} being the anhysteretic magnetization.

The effective magnetic field **H*eff*** is defined by the equation (10), where the inter-domain coupling parameter (α) quantifies the interaction between adjacent magnetic domains:

$$H_{eff} = H + \alpha M \tag{10}$$

The total magnetization of a material (***M***) comprises a reversible component (***M_{rev}***) and an irreversible one (M_{irr}), because of the elastic bending of magnetic domain walls and structural discontinuities within the material, respectively:

$$M = M_{rev} + M_{irr} \tag{11}$$

The irreversible component of the magnetization is given by the following differential equation:

$$\frac{dM_{irr}}{dt} = g(\frac{M_{rev}}{c\,k}\cdot\frac{dH_e}{dt})\frac{M_{rev}}{|M_{rev}|} \tag{12}$$

Where k is the pinning loss and $g = 1\ if\ dH/dt > 0$ and $g = -1\ if\ dH/dt < 0$.

The reversible part of the magnetization can be expressed with the following equation:

$$M_{rev} = \xi(M_{an} - M_{irr}) \tag{13}$$

and it represents a certain percentage (ξ) of the difference between the anhysteretic magnetization (***M_{an}***) and the irreversible magnetization (***M_{irr}***).

More specifically, ***M_{an}*** is described by the Langevin's function:

$$M_{an} = M_s(\coth\frac{|H_{eff}|}{a} - \frac{a}{|H_{eff}|})\frac{H_{eff}}{|H_{effe}|} \tag{14}$$

where a is proportional to the magnetic domain density and M_s is the saturation magnetization.

By combining the equations above, we can conclude that **H*eff*** represents the effective magnetic field within the magnetostrictive core and is defined by the following equation:

$$Heff = H + \alpha M + \frac{3\lambda_s}{\mu_0 M_s^2} S_{dev} M \tag{15}$$

where S_{dev} is the deviatoric part of the stress tensor. The final term, known as the "Villari effect," represents the contribution of mechanical stress to material magnetization. This term depends on the saturation magnetization *Ms*, the magnetostriction coefficient λ_s, and the deviatoric stress tensor.

Let us now consider our MENP model, characterized by a 25 nm thick piezoelectric barium titanate shell and a 90 nm-diameter magnetostrictive cobalt ferrite core. The magnetic moment as a function of the applied magnetic field for the cobalt ferrite core produced a typical hysteresis loop, as shown in the Figure S1 below.

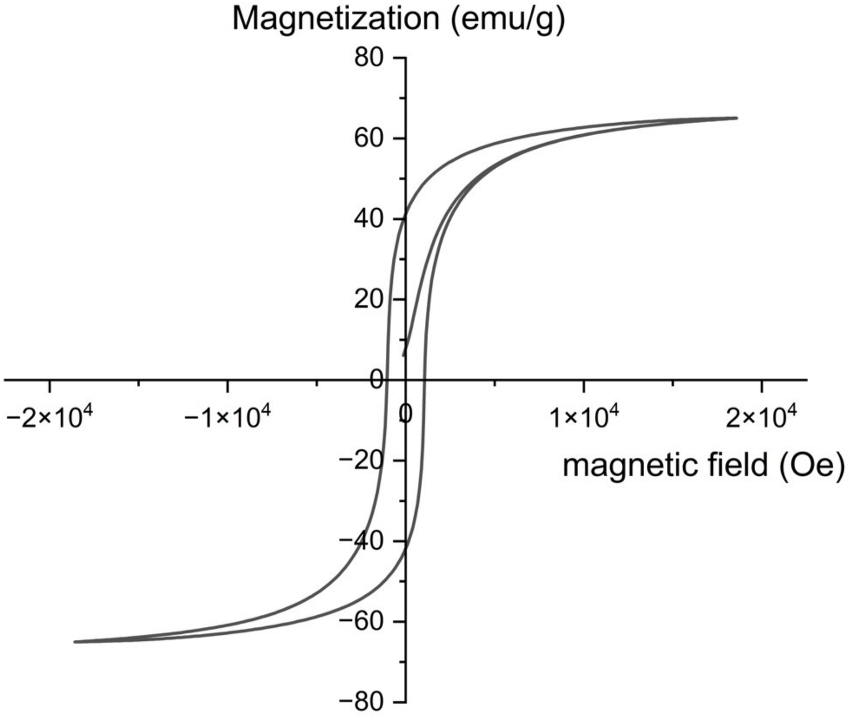

Figure S1: Magnetization loop of the cobalt ferrite core (90 nm of diameter).

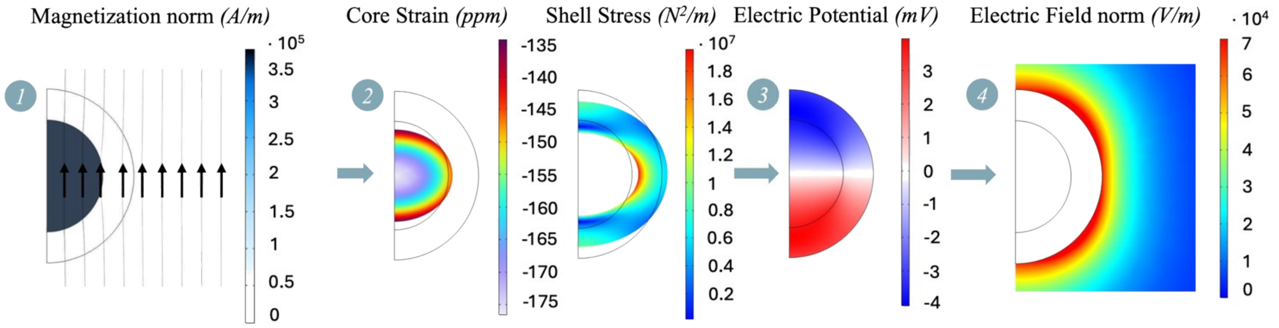

Figure S2: Static study results to validate the MENP baseline behavior, starting from magnetostriction in the cobalt ferrite core (1), passing through the core strain and shell stress (2) to the generation of an electric potential on the particle surface (3) and the electric field in its vicinity (4). Data refers to the case of the culture medium (CM) environment.

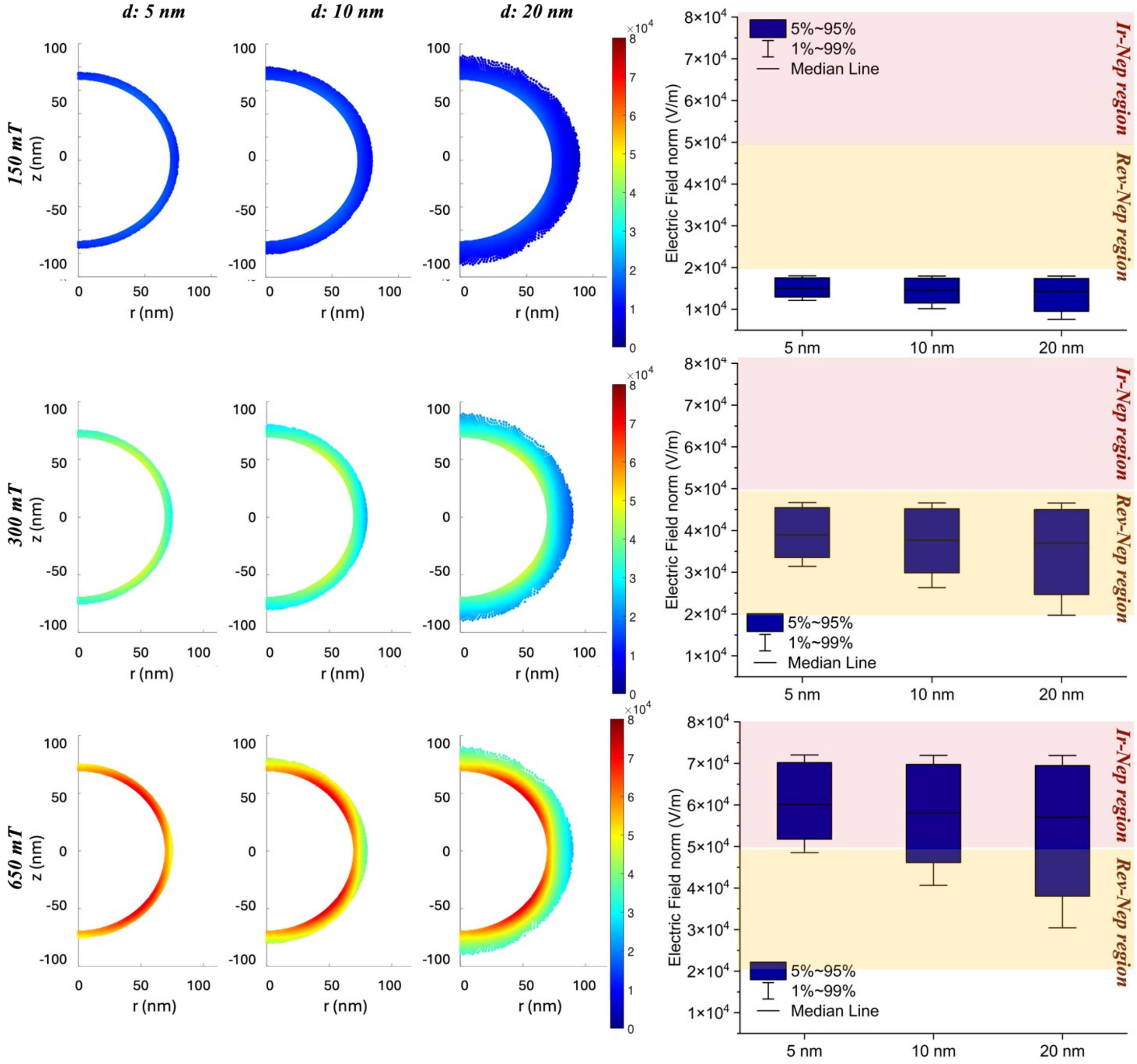

Figure S3: Left: Distributions of the electric field produced by various external H_{DC} fields ($H_{ext(1)}$ =150 mT, $H_{ext(2)}$ = 300 mT and $H_{ext(3)}$ = 650 mT) within culture medium surroundings, at gradually increasing distances from the nanoparticle outer shell (5 nm, 10 nm and 20 nm); right: statistical metrics box plot of the electric field distributions visualized on the left, reported in terms of median values, and percentiles. Data refers to the case of the CM environment.

Table S1: Statistical metrics in terms of median values, and 1st and 99th percentiles of E_{field} generated by $H_{ext(1)}$ (150 mT), $H_{ext(2)}$ (300 mT) and $H_{ext(3)}$ (650 mT). Data refers to the case of the CM environment.

	**$H_{ext(1)}$ = 150 mT**			**$H_{ext(2)}$ = 300 mT**			**$H_{ext(3)}$ = 650 mT**		
**(V/m)**	*5 nm*	*10 nm*	*20 nm*	*5 nm*	*10 nm*	*20 nm*	*5 nm*	*10 nm*	*20 nm*
Median	$1.50 \cdot 10^4$	$1.45 \cdot 10^4$	$1.43 \cdot 10^4$	$3.90 \cdot 10^4$	$3.77 \cdot 10^4$	$3.70 \cdot 10^4$	$6.01 \cdot 10^4$	$5.81 \cdot 10^4$	$5.71 \cdot 10^4$

99th percentile	1.80 · 10^4	1.80 · 10^4	1.79 · 10^4	4.67 · 10^4	4.66 · 10^4	4.66 · 10^4	7.21 · 10^4	7.19 · 10^4	7.19 · 10^4
1st percentile	1.21 · 10^4	1.02 · 10^4	0.76 · 10^4	3.14 · 10^4	2.63 · 10^4	1.97 · 10^4	4.85 · 10^4	4.06 · 10^4	3.04 · 10^4

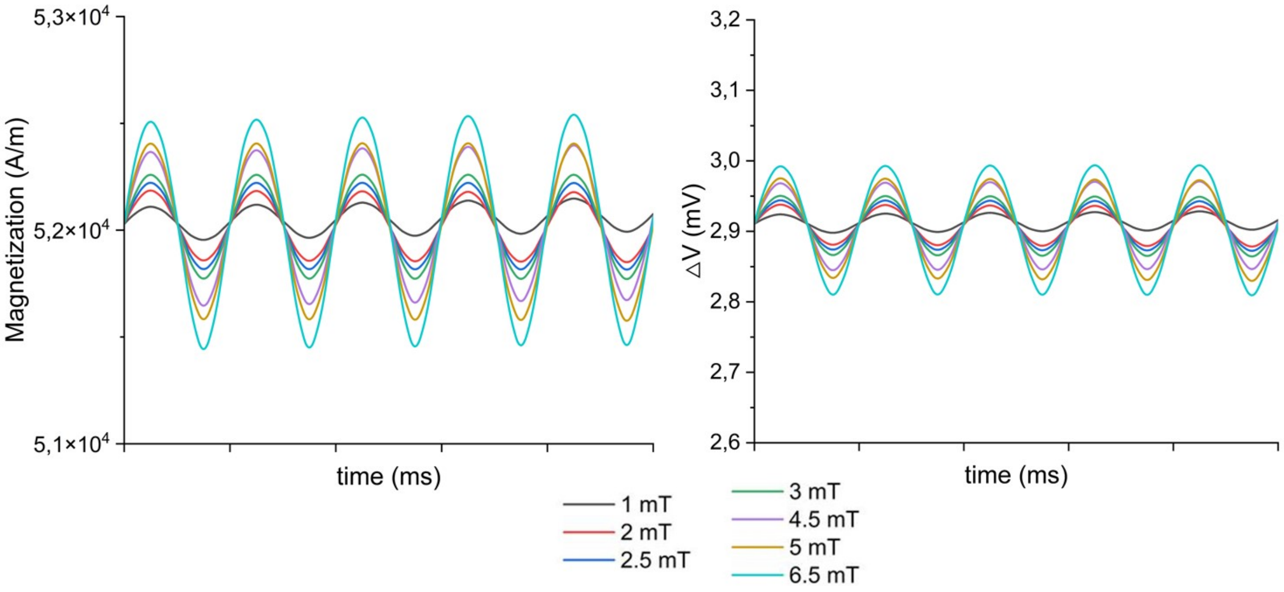

Figure S4: Time dependent study for the drug release step in the CM environment: MENP core magnetization behavior over time for different amplitudes of H_{AC} (on the left), and electric potential difference profiles over time for different H_{AC} (on the right).

Table S2: MENP core magnetization, electric potential difference and magnetoelectric coefficient values in relation to the increase in amplitude of the applied H_{AC} for the CM case scenario. Each parameter is calculated at the instant in which the peak of the H_{AC} sinusoidal wave is reached.

H_{AC} (Oe)	***Magnetization (A/m)***	***ΔV (mV)***	***α_{ME} (mV/cm*Oe)***
10	5.21 · 10^4	2.92	2.09 · 10^4
20	5.22 · 10^4	2.94	1.05 · 10^4
25	5.22 · 10^4	2.94	8.41 · 10^3
30	5.22 · 10^4	2.95	7.02 · 10^3
45	5.23 · 10^4	2.97	4.71 · 10^3
50	5.24 · 10^4	2.97	4.25 · 10^3
65	5.25 · 10^4	2.99	3.28 · 10^3

Theoretical Model of the Drug Delivery Phenomenon

The following equations report the mathematical model theorized by Stimphil *et al.*[3], where the nanoparticles magnetoelectric effect has been correlated with the displacement of the positive and negative charges involved in a hypothetical ionic drug-MENP bond. Considering a single MENP, the presence of an alternating magnetic field induces an electric dipole moment expressed as:

$$P = a_{ME,P} \cdot H \tag{16}$$

Where P is the electric dipole moment induced by an external magnetic field due to the ME effect, $\alpha_{ME,P}$ is the magnetoelectric coefficient and H is the external magnetic field. In this equation, $\alpha_{ME,P}$ is considered as the ratio between the polarization developed at the MENP surface and the external magnetic stimulus applied, and it is given in $[\frac{C}{m^2 \times Oe}]$. The polarization is linked to the electric field (E) according to equation (17):

$$P = \varepsilon \cdot E = \varepsilon \cdot \frac{\Delta V}{\emptyset_{MENP}} \tag{17}$$

Where $\varepsilon = \varepsilon_0(\varepsilon_r - 1)$ represents the electric permittivity of the system under study, expressed in terms of vacuum permittivity (ε_0) and the permittivity of the piezoelectric shell (ε_r), while ΔV stands for the potential difference developed across the MENP of diameter φ_{MENP}.

Therefore, the magnitude of the dipole charge surface density (σ_{MENP}) would be of the order of magnitude of:

$$\sigma_{MENP} \sim \pm a_{ME,P} \cdot H \tag{18}$$

Let us consider a hypothetical drug molecule linked at the MENP surface via an ionic bond. The phenomenon of drug release from the MENP could occur if the dipole charge surface density equals or exceeds the charge density involved in the original ionic bond, which can be defined as:

$$\sigma_{bond} \sim \frac{Q_{ionic}}{\pi \cdot {\emptyset_{MENP}}^2} \tag{19}$$

where Q_{ionic} is the displacement charge. Therefore, the threshold magnetic field amplitude (H_{th}) to break the hypothetical ionic drug-MENP bond can be derived according to the following expression:

$$H_{th} \sim \frac{Q_{ionic}}{\pi \cdot \emptyset_{MENP}{}^{2} \cdot \alpha_{ME,P}} \tag{20}$$

A correlation between the displacement charge (within the ionic bond) and the electrical potential difference (at the MENP surface) can be derived considering the following:

$$Q_{ionic} \sim H_{th} \cdot \pi \cdot \emptyset_{MENP}{}^{2} \cdot \alpha_{ME,P} \tag{21}$$

By expressing the magnetoelectric coefficient as $\alpha_{ME,P} = \varepsilon \cdot \frac{\Delta V}{\emptyset_{MENP}} \frac{1}{H_{th}}$ from expression 16 and 17, we obtain:

$$Q_{ionic} \sim H_{th} \cdot \pi \emptyset_{MENP}{}^{2} \cdot \varepsilon \cdot \frac{\Delta V}{H_{th} \cdot \emptyset_{MENP}} \sim \pi \cdot \varepsilon \cdot \emptyset_{MENP} \cdot \Delta V \tag{22}$$

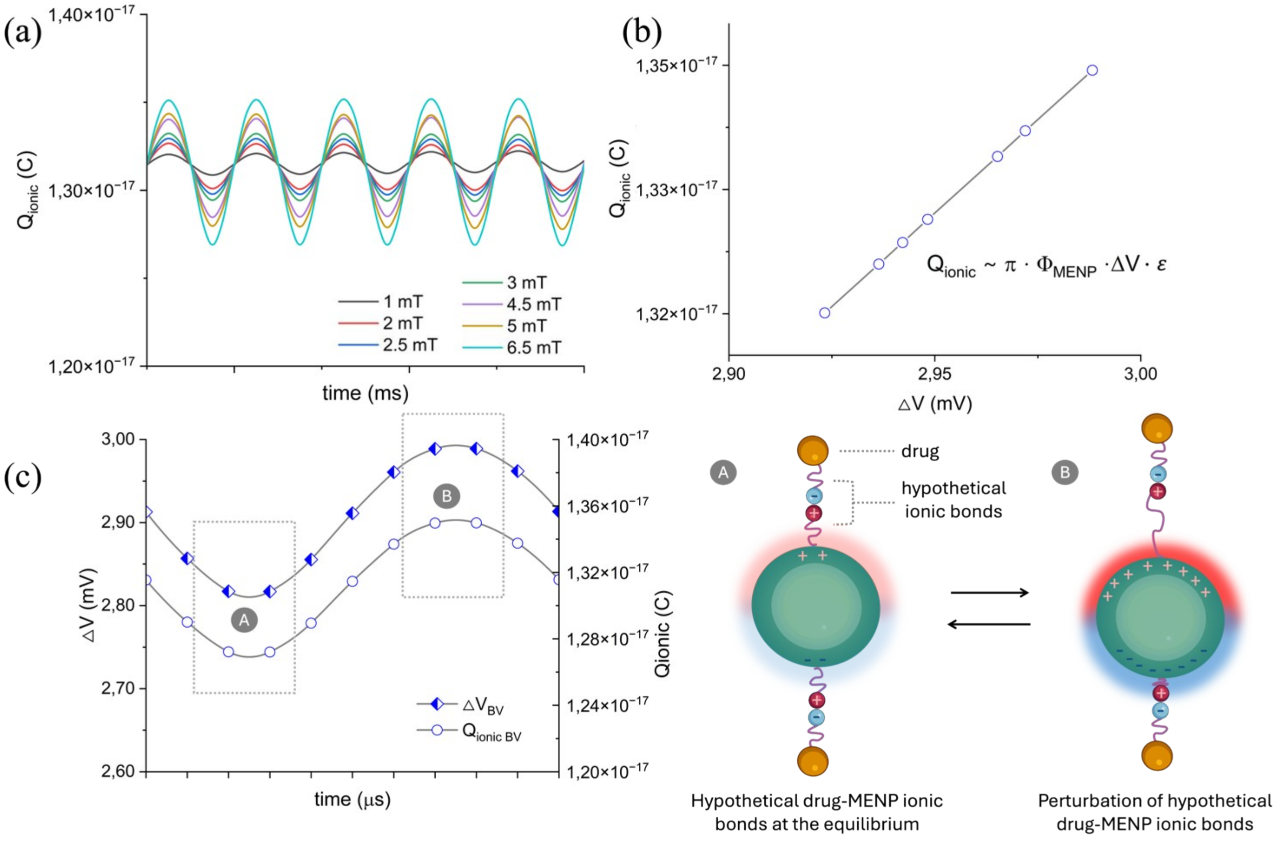

Figure S5: Ionic bond charge displacement parameter for the CM case scenario: (a) charge displacement in the hypothetical drug-MENP ionic bond profile over time for different H_{AC} amplitudes; (b) charge displacement in the hypothetical drug-MENP ionic bond values in relation to the various electric potential differences generated at the MENP surface at varying H_{AC} stimulations, considered at a specific time point;

(c) comparison between the trends of the charge displacement in the hypothetical drug-MENP ionic bond and the electric potential difference over a certain period of time. In (c), as a representative example, only the H_{AC} = 6.5 mT case was considered for the data presentation. Drug-MENP sketch: case *A* illustrates two hypothetical drug-MENP ionic bonds at the equilibrium, where the electric potential difference is at its minimum and so the average displacement of charges; case *B* represents a perturbed state of the ionic bonds, characterized by the movement of the positive and negative charges from their equilibrium positions, which causes bond length stretching and bond length contraction. In *A*, less intense red and blue colorations indicate lower ΔV at the MENP surface, while in *B* more intense blue and red shadows refer to higher ΔV.

References

1. Fiocchi, S. *et al.* Modeling of core-shell magneto-electric nanoparticles for biomedical applications: Effect of composition, dimension, and magnetic field features on magnetoelectric response. *PLoS One* **17**, (2022).

2. Marrella, A. *et al.* Magnetoelectric nanoparticles shape modulates their electrical output. *Front Bioeng Biotechnol* **11**, (2023).

3. Stimphil, E. *et al.* Physics considerations in targeted anticancer drug delivery by magnetoelectric nanoparticles. *Appl Phys Rev* **4**, (2017).
